# DNA-methylation-mediated activating of lncRNA SNHG12 promotes temozolomide resistance in glioblastoma

**DOI:** 10.1186/s12943-020-1137-5

**Published:** 2020-02-10

**Authors:** Chenfei Lu, Yutian Wei, Xiefeng Wang, Zhuoran Zhang, Jianxing Yin, Wentao Li, Lijiu Chen, Xiao Lyu, Zhumei Shi, Wei Yan, Yongping You

**Affiliations:** 1 Department of Neurosurgery, The First Affiliated Hospital of Nanjing Medical University, Nanjing, 210029 Jiangsu China; 2 Jiangsu Key Lab of Cancer Biomarkers, Prevention and Treatment, Jiangsu Collaborative innovation Center For Cancer Personalized Medicine, Nanjing Medical University, Nanjing, 211166 Jiangsu China; 3 Institute for Brain Tumors, Jiangsu Key Lab of Cancer Biomarkers, Prevention and Treatment, Jiangsu Collaborative Innovation Center for Cancer Personalized Medicine, Nanjing Medical University, Nanjing, 211166 Jiangsu China

**Keywords:** SNHG12, Temozolomide, Drug resistance, DNA methylation, Glioblastoma

## Abstract

**Background:**

Accumulating evidence shows that long noncoding RNAs (lncRNAs) are important regulator molecules involved in diverse biological processes. Acquired drug resistance is a major challenge in the clinical treatment of glioblastoma (GBM), and lncRNAs have been shown to play a role in chemotherapy resistance. However, the underlying mechanisms by which lncRNA mediates TMZ resistance in GBM remain poorly characterized.

**Methods:**

Quantitative reverse transcription PCR (qRT-PCR) and fluorescence in situ hybridization assays were used to detect small nucleolar RNA host gene 12 (SNHG12) levels in TMZ-sensitive and TMZ-resistant GBM cells and tissues. The effects of SNHG12 on TMZ resistance were investigated through in vitro assays (western blots, colony formation assays, flow cytometry assays, and TUNEL assays). The mechanism mediating the high expression of SNHG12 in TMZ-resistant cells and its relationships with miR-129-5p, mitogen-activated protein kinase 1 (MAPK1), and E2F transcription factor 7 (E2F7) were determined by bioinformatic analysis, bisulfite amplicon sequencing, methylation-specific PCR, dual luciferase reporter assays, chromatin immunoprecipitation assays, RNA immunoprecipitation assays, immunofluorescence, qRT-PCR, and western blot. For in vivo experiments, an intracranial xenograft tumor mouse model was used to investigate SNHG12 function.

**Results:**

SNHG12 was upregulated in TMZ-resistant cells and tissues. Overexpression of SNHG12 led to the development of acquired TMZ resistance, while knockdown of SNHG12 restored TMZ sensitivity. An abnormally low level of DNA methylation was detected within the promoter region of SNHG12, and loss of DNA methylation made this region more accessible to the Sp1 transcription factor (SP1); this indicated that methylation and SP1 work together to regulate SNHG12 expression. In the cytoplasm, SNHG12 served as a sponge for miR-129-5p, leading to upregulation of MAPK1 and E2F7 and endowing the GBM cells with TMZ resistance. Disinhibition of MAPK1 regulated TMZ-induced cell apoptosis and the G1/S cell cycle transition by activating the MAPK/ERK pathway, while E2F7 dysregulation was primarily associated with G1/S cell cycle transition. Clinically, SNHG12 overexpression was associated with poor survival of GBM patients undergoing TMZ treatment.

**Conclusion:**

Our results suggest that SNHG12 could serve as a promising therapeutic target to surmount TMZ resistance, thereby improving the clinical efficacy of TMZ chemotherapy.

## Background

Gliomas comprise the most common primary brain tumor and more than half of these are glioblastoma tumors, the most malignant of all the brain tumors. Even with the most aggressive treatment, the median survival of glioblastoma (GBM) patients is still less than 15 months [1, 2]. Temozolomide (TMZ) is a second-generation oral alkylating agent that readily passes through the blood-brain barrier, and it is the standard first-line chemotherapy for the clinical treatment of glioblastoma [3, 4]. However, improvements in the prognosis for these brain tumors are slow to fruition, owing to therapeutic resistance and postoperative tumor recurrence [5]. Thus, elucidating the underlying mechanisms of TMZ resistance and exploring reliable biomarkers to predict TMZ response in GBM patients are urgently needed.

Long non-coding RNA (lncRNA) is a class of heterogeneous RNA with a length of more than 200 nucleotides. LncRNAs play important roles in tumorigenesis, through effects on dose compensation, epigenetic regulation, cell cycle regulation, and drug resistance [6, 7]. Epigenetic changes have been identified as one of the hallmarks of tumorigenesis [8], and emerging evidence shows that epigenetic regulation is one of the main mechanisms regulating lncRNA expression and tissue specificity [9, 10]. However, the epigenetic regulation of lncRNAs and the subsequent effect on tumor progression, especially in terms of acquired chemoresistance, remain largely unknown. LncRNAs have been propounded to act as competitive endogenous RNA (ceRNA) that compete for microRNA (miRNA) binding, thereby playing a significant role in gene regulation [11, 12]. The ceRNA network participates in the mediation of postoperative treatment resistance in some cancers but its role in TMZ resistance is rarely reported.

In this study, we investigated the role of lncRNA SNHG12 (small nucleolar RNA host gene 12) in acquired TMZ resistance in GBM and the effect of epigenetic regulation on its abnormal expression. Our results showed that SNHG12 is epigenetically activated by DNA methylation at the CpG islands within its promoter region. SNHG12 regulates the MAPK/ERK signaling pathway and G1/S cell cycle transition by competitively binding to miR-129-5p, which, in turn, modulates TMZ resistance in GBM cells.

## Methods

### Patients and specimens

The 40 primary GBM specimens and 20 recurrent GBM specimens used in this study were obtained by surgical resection from patients undergoing TMZ chemotherapy (Department of Neurosurgery, the First Affiliated Hospital of Nanjing Medical University, Nanjing, China). The experiment has passed ethical review by the medical ethics committee of the First Affiliated Hospital of Nanjing Medical University (Ethics number: 2019-SR-479). The diagnosis of glioma was confirmed by pathologists. Detailed patient information is presented in Additional file 1: Table S1.

### Public data collection

Microarray datasets and their associated clinical information were downloaded from the Chinese Glioma Genome Atlas (CGGA; http://www.cgga.org.cn) and the Rembrandt microarray database (http://caintegrator.nci.nih.gov/rembrandt/), and raw microarray data from the Gene Expression Omnibus (GEO) databases were used to detect differential expression of SNHG12 (https://www.ncbi.nlm.nih.gov/geo/; including GSE4290, GSE7696, GSE15824, GSE50161, GSE59612, and GSE104267).

### Cell lines and cell culture

The human embryonic kidney (HEK) 293 T cell line was purchased from the Chinese Academy of Sciences Cell Bank (Shanghai, China). N3 patient-derived cells were obtained from the China National Clinical Research Center for Neurological Diseases, Beijing Tian Tan Hospital. Six drug-related cell lines (Pri GBM, Rec GBM, N3S, N3T3rd, U251, and U251T3rd) were as described in our previous report [13]. All cells were cultured in high-glucose Dulbecco’s modified Eagle’s medium (DMEM) supplemented with 10% fetal bovine serum (FBS) at 37 °C with 5% CO_2_.

### RNA extraction and quantitative real-time PCR assays

Total RNA was extracted from tissues and cell lines with the TRIzol reagent (Invitrogen, CA, USA) according to the manufacturer’s protocol. The nuclear and cytoplasmic fractions were separated with the PARIS Kit (Invitrogen, CA, USA). cDNA was synthesized with the PrimeScript RT Reagent Kit (Takara, Nanjing, China). Real-time quantitative PCR (qRT-PCR) analyses were performed with the SYBR Green Premix Ex Taq (Takara, Nanjing, China). The total RNA levels were normalized with GAPDH. U6 snRNA (small nuclear RNA) was used as the miRNA internal control. Relative RNA expression levels were measured with the ABI 7500 real-time PCR system (Applied Biosystems, Foster City, CA, USA). Primer sequences are shown in Additional file 2: Table S2. The relative quantitative value for each gene was determined as 2^−∆∆CT^.

### Western blot assay

Western blot analysis was performed by following the manufacturer’s protocol as previously described [13]. Antibodies used are listed in Additional file 3: Table S3.

### Plasmid construction and cell transfection

Lipofectamine 2000 (Invitrogen) was used to transfect the siRNAs, miRNA mimics, and plasmids into the GBM cells. All small interfering RNA (siRNA) and short hairpin RNA (shRNA) sequences designed for specific targets are listed in Additional file 4: Table S4. We synthesized full-length complementary cDNAs of human SNHG12, SP1, and MAPK1, and cloned these cDNAs into the expression vector pcDNA3.1 (Invitrogen). SNHG12 shRNAs and the negative control RNA (sh-Ctrl) were designed and synthesized by Genechem (Shanghai, China). Rec GBM and N3T3rd cells were used to establish stable cell lines and selected with puromycin at 48 h after injection. SP1 siRNA, MAPK1 siRNA, E2F7 siRNA, miR-129-5p mimics, and the miR-129-5p inhibitor were purchased from Genechem.

### Methylation-specific PCR and bisulfite sequencing

Genomic DNA was extracted from GBM and normal tissues with a QIAamp DNA Mini Kit (Qiagen). The purified DNA was exposed to bisulfite with an EpiTect Bisulfite Kit (Qiagen) according to the manufacturer’s protocol. Using the GeneAmp PCR System 2700 (Applied Biosystems, Grand Island, NY, USA), the methylation-specific PCR (MSP) of bisulfite-transformed DNA was carried out with a nested, two-stage PCR method. Amplified PCR products were separated by 3% agarose gel electrophoresis and visualized with GelRed (Vazyme, Nanjing, China). For bisulfite-sequencing PCR (BSP), bisulfite-converted genomic DNA was amplified using specific BSP primers and the sequencing library was prepared with the VAHTS Turbo DNA Library Prep Kit (Vazyme, Nanjing, China). The specific primers used for MSP and BSP are listed in Additional file 2: Table S2.

### Fluorescence in situ hybridization (FISH) and immunohistochemistry (IHC) analysis

FISH analysis was performed on human tissues and GBM cells as previously described [14]. IHC was performed on mice xenogeneic tumor tissues as previously described [14].

### Flow cytometry analysis of cell cycle transitions and apoptosis

For cell cycle analysis, the cells were harvested 24 h after serum starvation and fixed overnight in 70% ethanol at 4 °C. Cells were incubated with propidium iodide (PI) staining solution before flow cytometry detection. For apoptosis analysis, the cells were stained with PI and Annexin V-FITC according to the manufacturer’s instructions (Roche, Basel, Switzerland).

### TUNEL assay

For the terminal deoxynucleotidyl transferase (TdT) dUTP nick-end labeling (TUNEL) assays, GBM cells were fixed in 4% paraformaldehyde for 15 min. Cells were then stained with the In Situ Cell Death Detection Kit, POD (Roche, Switzerland) according to the manufacturer’s protocol. Images were acquired with a Nikon ECLIPSE E800 fluorescence microscope.

### CCK-8 and colony formation assays

GBM cells were seeded in 96-well plates and cell viability was evaluated with the Cell Counting Kit 8 (Dojindo, Shanghai, China). Absorbance was measured (OD value) at a wavelength of 450 nm.

For the colony formation assay, cells were seeded in six-well plates and cultured for 11 days with or without TMZ treatment. The resulting colonies were washed twice with PBS, fixed with 4% formaldehyde for 10 min, and stained with 0.1% crystal violet for 30 min.

### Luciferase reporter assays

For the luciferase reporter assay at the SNHG12 promoter region, HEK293T cells were co-transfected with luciferase reporter packaging the sequence of SNHG12 promoter region and the empty vector, or the TFAP2A, TFAP4, SP1, STAT1 or IKZF1 plasmid (Genechem, Shanghai, China). The promoter region only contained the P1, P2 or P3 regions and these sequences were synthesized and cloned into the pGL3-basic luciferase reporter vector (Promega, Madison, USA). For miRNA target gene luciferase reporter assays, SNHG12, MAPK1, and E2F7 wild-type sequences with potential miR-129-5p-binding sites or mutants of each binding site were synthesized and co-transfected into N3T3rd and Rec GBM cells. All luciferase activities were measured with the Dual Luciferase Reporter Assay System (Promega) and normalized to *Renilla* luciferase activity.

### Immunofluorescence

Cells were fixed in 4% paraformaldehyde for 15 min and then permeabilized with 0.25% Triton X-100 (Beyotime, Shanghai, China) at room temperature. The cells were blocked with 1% bovine serum albumin for 20 min and then incubated with primary antibody at 4 °C overnight. After washing with PBS three times, the cells were incubated with goat anti-rabbit IgG secondary antibodies (FITC Green goat anti-rabbit; Molecular Probes, Shanghai, China) for 1 h at room temperature. The nucleic acids were stained with DAPI (Sigma-Aldrich, Shanghai, China). The images were captured with a Nikon ECLIPSE E800 fluorescence microscope.

### RNA immunoprecipitation (RIP)

The RIP experiments were performed with a Magna RIP RNA-Binding Protein Immunoprecipitation Kit (Millipore, Billerica, MA, USA) according to the manufacturer’s protocol. GBM cell lysates were prepared and incubated with RIP buffer containing magnetic beads conjugated with human anti-argonaute-2 (anti-Ago2) antibody (Cat. ab32381; Abcam). Normal mouse IgG (Cat. 12–371; Millipore) functioned as the negative control. The RNA fraction precipitated by RIP was analyzed by qPCR.

### Chromatin immunoprecipitation (ChIP)

ChIP assays were performed with an EZ-ChIP Kit (Millipore) according to the manufacturer’s instructions. Briefly, GBM cells were cross-linked with 1% formaldehyde for 10 min and then quenched with glycine. Cell lysates were then sonicated to generate chromatin fragments and then immunoprecipitated with H3K4me3 antibody (Cat. 39,915; Active Motif, Shanghai, China), H3K27me3 antibody (Cat. 39,155; Active Motif, Shanghai, China) or SP1 antibody (Cat. 9389; Cell Signaling Technology), and IgG antibody (Cat. 12–371; Millipore) was used as the negative control. The ChIP primer sequences are listed in Additional file 2: Table S2.

### In vivo xenograft model

Four-week-old female BALB/c nude mice were purchased from the Experimental Animal Center of Nanjing Medical University. To establish the intracranial tumor model, 2.5 × 10^5^ recurrent GBM cells were separately implanted stereotactically into the nude mice brain. After surgery, the mice were treated with or without TMZ by oral gavage at 1 week (66 mg/kg per day for 5 days). Bioluminescence imaging (IVIS Spectrum; PerkinElmer, USA) was used to confirm intracranial tumor formation and tumors were measured each week. The procedures used for animal treatments and experiments conformed with the Guide for the Care and Use of Laboratory Animals and this study was approved by the Nanjing Medical University Animal Experimental Ethics Committee.

### Statistical analysis

All statistical analyses were performed with GraphPad software version 7.0 (GraphPad Software, San Diego, CA, USA) or IBM SPSS Statistics 23.0 software (SPSS, Chicago, IL, USA). The significance of the differences between groups was estimated with Student’s *t* test, chi-square test or one-way analysis of variance (ANOVA) as appropriate. The Kaplan–Meier method with the log-rank test was used to calculate the overall survival (OS) rate for comparison between different groups. The correlations between variables were analyzed with the Pearson correlation coefficient. GO and KEGG pathway analyses were performed on the DAVID website (https://david.ncifcrf.gov/). DIANA tools (http://diana.imis.athena-innovation.gr/) and miRcode (http://www.mircode.org/) were used to predict lncRNA-targeting miRNAs. All results are shown as the mean ± standard error of the mean (SEM) of three independent experiments. Statistical significance was considered to be represented by a value of *p* < 0.05. Additional file 5: Table S5-7.

## Results

### SNHG12 is highly expressed in TMZ-resistant GBM cell lines and tissues after TMZ treatment

**Fig. 1:**
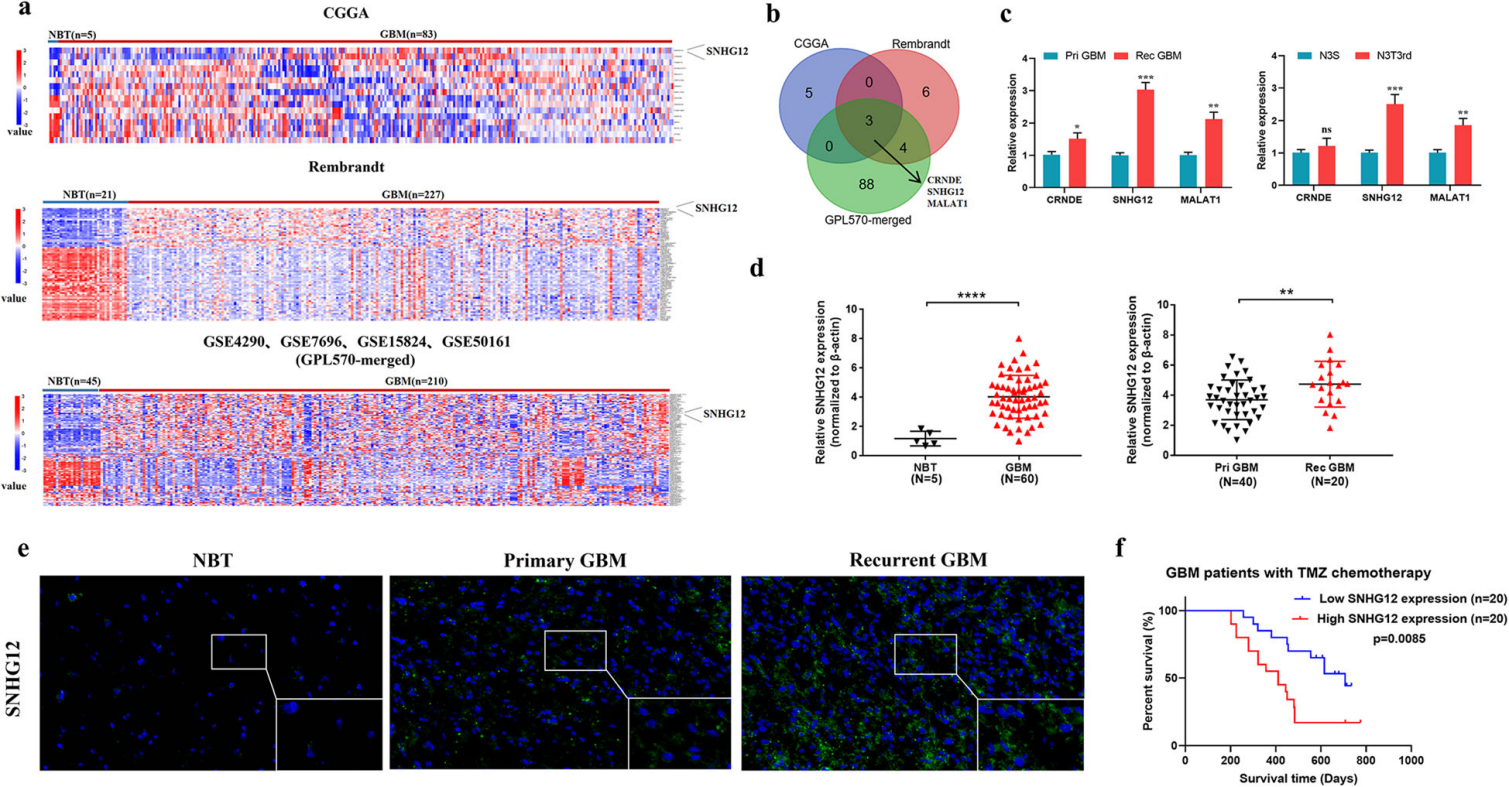
SNHG12 is up-regulated in TMZ-resistant GBM tissues and cell lines. **a** Hierarchical clustering analysis of lncRNAs that were differentially expressed in glioma samples. **b** Overlap of lncRNAs in the CGGA, Rembrandt, and GEO data sets **c** The expression level of SNHG12 in TMZ-resistant and TMZ-sensitive cells. **d** Relative expression of SNHG12 in primary and recurrent GBM tissues. The SNHG12 expression was normalized to β-actin. **e** FISH analysis of SNHG12 expression in normal brain tissues, primary GBM tissues and recurrent tissues. Scale bar = 50 μm. **f** Kaplan-Meier survival analysis of OS in GBM patients with TMZ chemotherapy (*n* = 40, *P* = 0.0085). Data are presented as the mean ± SEM from three independent experiments. Significant results were presented as NS non-significant, *P<0.05, **P<0.01, ***P<0.001, ****P<0.0001

We firstly analyzed six raw microarray datasets downloaded from glioma genome atlas public databases such as CGGA, Rembrandt, and GEO (GSE4290, GSE7696, GSE15824, and GSE50161; all used the GPL570[HG-U133_Plus_2] platform). To obtain differentially expressed lncRNAs, signal data were normalized (a group or paired t-test was used to verify statistical significance according to the experimental design). Heatmaps were then generated to show the expression levels of the up- and down-regulated lncRNAs (Fig. 1a). We particularly focused on the overexpressed lncRNAs because these lncRNAs could be more readily used as clinical therapeutic targets or potential biomarkers. SNHG12, CRNDE, and MALAT1 were found to be highly expressed in all six datasets (Fig. 1b).

Recent studies have shown that lncRNA-regulated TMZ-resistance mechanisms in GBM can be key nodes of therapeutic intervention. For example, lncRNA MALAT1 has the ability to promote TMZ resistance in GBM [15]. To determine whether lncRNAs play a crucial role in acquired TMZ-resistance, qRT-PCR of three paired TMZ-sensitive and TMZ-resistant GBM cell lines was used to detect the expression levels of three lncRNAs after TMZ treatment. SNHG12 expression was significantly up-regulated in all of the TMZ-resistant cells (Fig. 1c and Additional file 6: Figure S1a). These cells revealed poor response to TMZ as illustrated by an increased half-maximal inhibitory concentration (IC_50_) and enhanced proliferation ability when undergoing TMZ treatment (Additional file 6: Figure S1b–c). In addition, a high level of SNHG12 was found in two other GEO datasets (GSE59612 and GSE104267) and SNHG12 expression was found to be relatively high in the recurrent GBM tissues analyzed in this study compared with the primary GBM tissues in GSE7696 (this dataset describes samples of recurrent GBM tissues after receiving TMZ treatment) (Additional file 6: Figure S1d). Next, we measured SNHG12 levels in five normal brain tissues, 40 primary GBMs, and 20 recurrent GBMs (insensitive to TMZ treatment) by qRT-PCR. As shown in Fig. 1d, SNHG12 was significantly up-regulated in the GBMs compared with normal tissues. Furthermore, SNHG12 expression was markedly increased in recurrent GBM tissues compared with primary GBM tissues. Similar results were also observed with FISH (Fig. 1e).

Next, Kaplan–Meier analysis was used to determine whether SNHG12 expression levels in the GBM tissues were associated with clinical response to TMZ therapy. Survival analysis of the CGGA cohort revealed that a higher SNHG12 level was associated with poor overall survival (OS) in GBM patients (Additional file 6: Figure S1e). Moreover, Kaplan–Meier analysis of our cohort showed that patients with low SNHG12 expression exhibited a superior OS after TMZ therapy, while patients with high SNHG12 expression exhibited poor response to TMZ therapy (Fig. 1g). Taken together, these results suggest that lncRNA SNHG12 is upregulated in TMZ-resistant GBM cell lines and tissues, pointing to a possible relationship between SNHG12 and acquired TMZ resistance.

### Knockdown of SNHG12 restores TMZ sensitivity in TMZ-resistant GBM cells in vitro

**Fig. 2:**
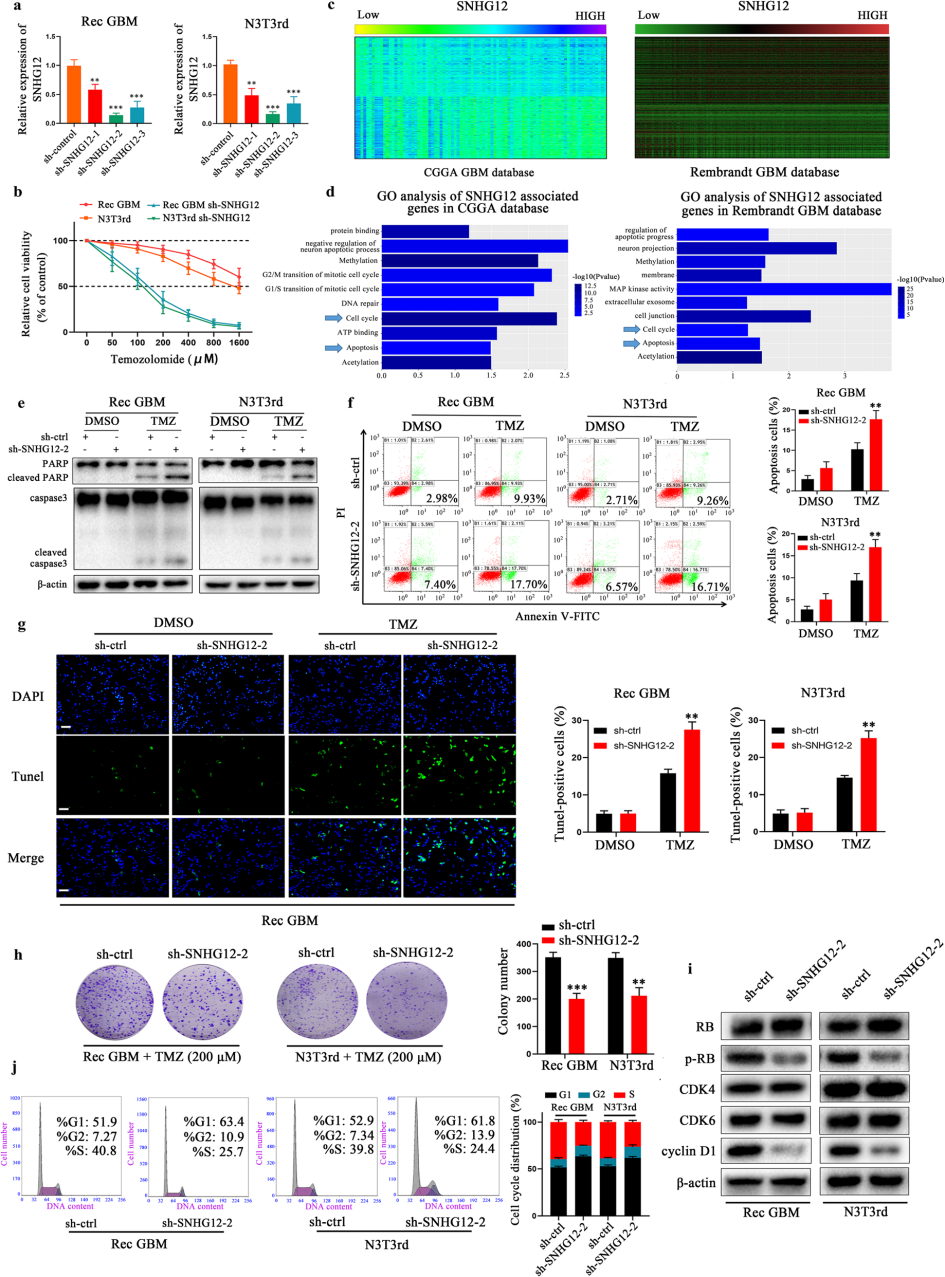
Knockdown of SNHG12 restores TMZ sensitivity in TMZ-resistant cells in vitro*.***a** qRT-PCR analysis of SNHG12 expression in sh-control, sh-SNHG12–1, sh-SNHG12–2, sh-SNHG12–3 treated TMZ-resistant cells. **b** CCK-8 assay analysis revealed the effect of SNHG12 knockdown on the TMZ-resistant cells after TMZ treatment at the indicated concentrations for 48 h. **c** Heatmaps of SNHG12-associated genes in 83 and 227 glioblastoma tissues sorted by the level of SNHG12 expression in CGGA and Rembrandt data sets. **d** GO analyses were performed using the SNHG12 associated genes in CGGA and Rembrandt data sets. **e** Western blot test of caspase-3 and PARP in TMZ-resistant cells treated with vehicle control or TMZ (200 μM) for 48 h. β-actin was used as the loading control. **f** Flow cytometric analysis revealing the effect of SNHG12 knockdown on the apoptosis of TMZ- resistant cells with or without TMZ treatment (200 μM, 48 h). **g** TUNEL analysis of SNHG12 knockdown cells or vehicle control with or without TMZ treatment (200 μM, 48 h). Scale bar = 50 μm. **h** Colony formation assays of SNHG12 knockdown or vehicle control TMZ-resistant cells with or without TMZ treatment (200 μM, 48 h). **i** Knockdown of SNHG12 decreased the levels of p-Rb, cyclin D1 but not CDK4 and CDK6 in TMZ-resistant cells. β-actin was used as the loading control. **j** The cell cycle distribution was analyzed by flow cytometric analysis in TMZ-resistant cells transfected with sh-ctrl or sh-SNHG12–2. Data are presented as the mean ± SEM from three independent experiments. Significant results were presented as NS non-significant, **P<0.01, ***P<0.001

To investigate the functional role of SNHG12 in acquired TMZ resistance, three independent SNHG12 shRNAs were transduced into TMZ-refractory Rec GBM cells and N3T3rd cells (TMZ-resistant cell lines; Additional file 6: Figure S1b–c), and SNHG12 expression was detected by qRT-PCR (Fig. 2a). Among these, Sh-SNHG12–2 was selected for subsequent experiments as it exhibited the strongest knockdown efficiency. Knockdown of SNHG12 in TMZ-resistant cells significantly decreased cell viability (Fig. 2b). Correlation analysis was performed on 83 and 227 samples with GBM mRNA datasets from the CGGA and Rembrandt databases, respectively, and a cluster (│r│ > 0.4, *P* < 0.05) of SNHG12-associated genes was obtained (Fig. 2c). Next, Gene Ontology (GO) analysis of these genes was conducted. We found that SNHG12-associated gene expression profiles were primarily enriched during cell cycle transitions and apoptosis (Fig. 2d). Transduction of SNHG12 shRNA into Rec GBM cells and N3T3rd cells markedly activated caspase-3 and cleavage of its substrate PARP (Fig. 2e), along with a higher level of cell apoptosis (Fig. 2f-g and Additional file 7: Figure S2a). Moreover, colony formation assays revealed that clonogenic survival was significantly decreased in SNHG12 knockdown Rec GBM cells and N3T3rd cells after TMZ treatment (Fig. 2h). SNHG12 depletion decreased phosphorylated Rb (p-Rb) and cyclin D1 levels (Fig. 2i), which was substantiated by G1/S cell cycle arrest as analyzed by flow cytometry (Fig. 2j). Together, these results suggest that inhibition of SNHG12 restores TMZ sensitivity in TMZ-resistant cells.

### SNHG12 overexpression confers resistance to TMZ

**Fig. 3:**
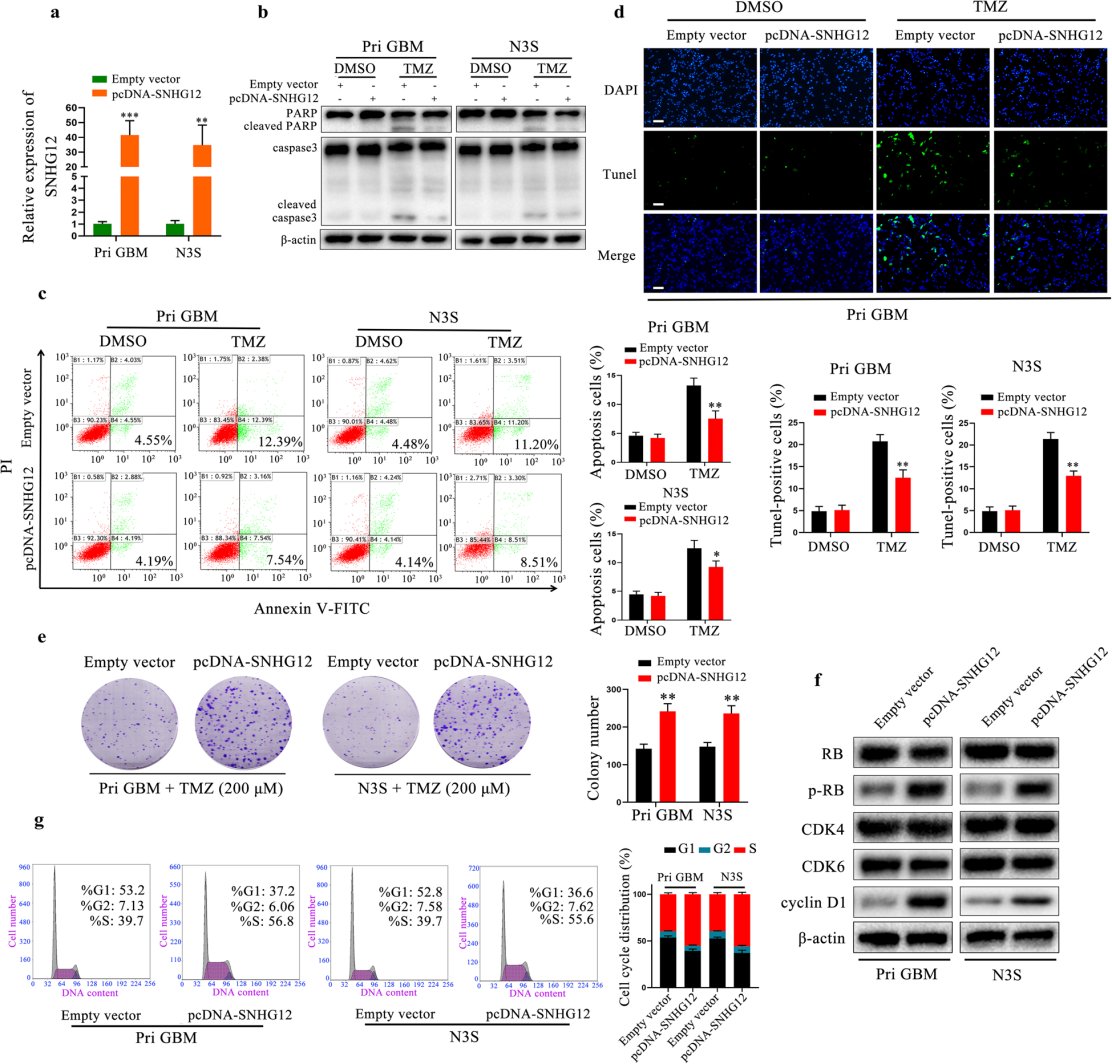
SNHG12 overexpression confers TMZ resistance. **a** qRT-PCR analysis of SNHG12 expression in Pri GBM and N3S cells transfected with empty vector or pcDNA-SNHG12. **b** Western blot analysis of caspase-3 and PARP in TMZ-sensitive cells with or without TMZ treatment (200 μM, 48 h). β-actin was used as the loading control. **c** Flow cytometric analysis revealing the effect of SNHG12 overexpression on the apoptosis of TMZ-sensitive cells with or without TMZ treatment (200 μM, 48 h). **d** TUNEL analysis of SNHG12 overexpression cells or empty vector with or without TMZ treatment (200 μM, 48 h). Scale bar = 50 μm. **e** Colony formation assays of SNHG12 overexpression or empty vector TMZ-sensitive cells with or without TMZ treatment (200 μM, 48 h). **f** Overexpression of SNHG12 increased the levels of p-Rb, cyclin D1 but not CDK4 and CDK6 in TMZ-sensitive cells. **g** The cell cycle distribution was analyzed by flow cytometric analysis in TMZ-sensitive cells transfected with empty vector or pcDNA-SNHG12. Data are presented as the mean ± SEM from three independent experiments. Significant results were presented as NS non-significant, *P<0.05, **P<0.01

Given that knockdown of SNHG12 was able to restore TMZ sensitivity in TMZ-resistant cells, we next investigated whether gain of SNHG12 could confer TMZ resistance. For this purpose, SNHG12 was overexpressed in N3S and Pri GBM cells (TMZ-sensitive cell lines, Additional file 6: Figure S1b–c and Fig. 3a) and we tested the apoptotic rate of these TMZ-sensitive cells following transduction with either pcDNA-SNHG12 or an empty vector. As shown in Fig. 3b–d and Additional file 7: Figure S2b, a marked decrease in the number of apoptotic cells was observed in these cells along with the co-dependent reduction of caspase-3 activity and cleavage of its substrate PARP compared with the control cells. These findings indicated that the overexpression of SNHG12 reversed TMZ-induced apoptosis. Next, we investigated whether gain or loss of SNHG12 could exert an influence on cell cycle distribution. Pri GBM and N3S cells transfected with the empty vector showed a marked decrease in colony-forming ability after TMZ treatment, whereas the growth inhibitory effects of TMZ treatment were partly reversed by SNHG12 overexpression (Fig. 3e). Overexpression of SNHG12 increased the levels of the cyclin D1 protein and phosphorylated Rb (Fig. 3f). In agreement with this finding, overexpression of SNHG12 clearly resulted in cell cycle redistribution with a significant decrease in the number of cells in the G1 phase (Fig. 3g). Hence, we concluded that SNHG12 enhanced the cell proliferation and TMZ resistance of GBM cells by promoting the G1/S cell cycle transition and inhibiting cell apoptosis.

### DNA methylation and SP1 regulate the expression of SNHG12

**Fig. 4:**
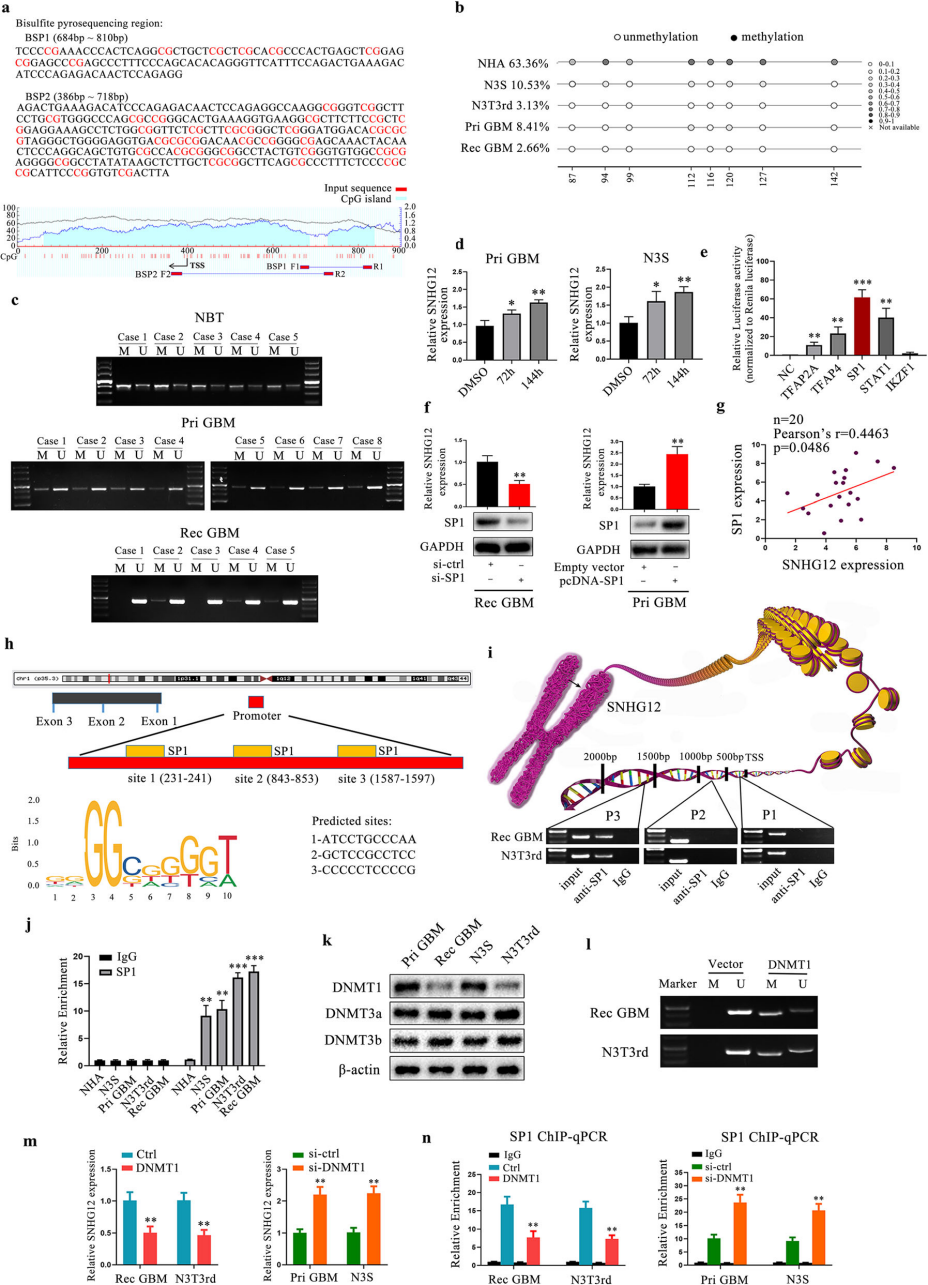
DNA methylation and SP1 are involved in the activation of SNHG12. **a** Schematic representation of the CpG islands and bisulfite sequencing; Magenta words, CG sites for bisulfite sequencing; Red region, input sequence; Blue region, CpG islands; BSP1 F1 and R1, BSP2 F1 and R1, bisulfite forward primer and reverse primer. **b** Bisulfite genomic sequencing was performed to examine methylation status of CpG island 1 at the promoter region of SNHG12 in NHA, Pri GBM, Rec GBM, N3S, N3T3rd cells. **c** MSP analysis was performed to examine methylation status of CpG island 1 at the promoter region of SNHG12 in normal brain tissues, primary GBM tissues and recurrent GBM tissues. **d** qRT-PCR analysis detecting the SNHG12 levels in Pri GBM and N3S cells after the treatment with 5-azacytidine for 72 h and 144 h. **e** The luciferase reporter plasmids carrying SNHG12 promoter region were co-transfected into HEK293T cells with five transcription factor plasmids, respectively. Relative luciferase activity in HEK293T cells were determined. **f** The SNHG12 levels were detected in Rec GBM and Pri GBM cells either stably expressing SP1 or with SP1 depleted. **g** The correlation between SP1 and SNHG12 in GBM tissues was analyzed. **h** Predicted SP1-binding sites in the promoter region of SNHG12. **i** ChIP-PCR assay of the enrichment of SP1 on the SNHG12 promoter region. **j** ChIP analysis for the detection of SP1 binding to the promoter region of SNHG12. **k** DNMT profiles using western blotting**. l** MSP analysis of CpG island 1 after DNMT1 overexpression. **m** qRT-PCR analysis of SNHG12 expression after DNMT1 overexpression or knockdown. **n** ChIP-PCR assay of the enrichment of SP1 on the SNHG12 promoter region after DNMT1 overexpression or knockdown. Data are presented as the mean ± SEM from three independent experiments. Significant results were presented as NS non-significant, *P<0.05, **P<0.01, ***P<0.001

We next explored the mechanisms underlying the high expression of SNHG12 in TMZ-resistant cells and tumor tissues after TMZ treatment. Firstly, with the use of the UCSC Genome Bioinformatics Site (http://genome.ucsc.edu/), we identified CpG islands together with high enrichment and overlapping H3K4me1 and H3K4me3 peaks within the promoter region of SNHG12 (Additional file 8: Figure S3a), indicating a potential relationship between SNHG12 expression and DNA methylation. Abnormal DNA methylation has been observed in many cancers, and this epigenetic disruption may lead to abnormal lncRNA expression in tumor tissues [16, 17]. Using methylation analysis software (http://www.urogene.org/methprimer/), we found two CpG islands in the promoter region of SNHG12, which we termed BSP1 and BSP2. Two specific primer sets were designed to detect changes in the DNA methylation levels of these two CpG islands (Fig. 4a). Bisulfite sequencing (BSP) analysis detected decreased levels of DNA methylation around the transcription start sites (TSS) of SNHG12 in TMZ-resistant cells compared to TMZ-sensitive cells and normal human astrocytes (NHA) cells (Fig. 4b). This decrease was observed at the BSP1 site but not at the BSP2 site (Additional file 8: Figure S3b). Next, using methylation-specific PCR (MSP), we analyzed the hypomethylation level within the promoter region of SNHG12 in recurrent GBM tissues (insensitive to TMZ treatment) compared to primary GBM tissues and normal brain tissues (Fig. 4c and Additional file 8: Figure S3c). After treatment with the DNA demethylating drug azacytidine, SNHG12 expression increased in Pri GBM cells and N3S cells, indicating that DNA demethylation increases SNHG12 expression (Fig. 4d).

Several studies have shown that transcription factors play an important role in regulating the expression of lncRNAs and DNA-methylated genes [18, 19]. We, therefore, used the JASPAR database (http://jaspar.genereg.net/) to perform bioinformatic analysis of the promoter region of SNHG12 to predict potential binding sites for transcription factors. Dual luciferase reporter assays were then used to detect the binding activity of the transcription factors among the top five predictions. HEK293T cells were transfected with a luciferase plasmid containing the promoter region of SNHG12 together with plasmids containing either the individual transcription factors or the control sequence. SP1 showed maximum luciferase activity (Fig. 4e) and depletion of SP1 decreased SNHG12 expression, while overexpression of SP1 increased SNHG12 levels (Fig. 4f and Additional file 8: Figure S3d). Furthermore, the expression levels of SP1 and SNHG12 were positively correlated in 20 recurrent GBM tissues, which was consistent with the analysis of CGGA and Rembrandt primary GBM tissue data (Fig. 4g and Additional file 8: Figure S3e). Next, bioinformatic analysis of the promoter region of SNHG12 predicted three potential binding sites for SP1 (Fig. 4h). A chromatin immunoprecipitation assay (CHIP) found enrichment at Site 3 (containing the BSP1 sequence) in TMZ-resistant cells (Fig. 4i). Furthermore, ChIP assays revealed that the enrichment of SP1 at Site 3 of SNHG12 was significantly increased in TMZ-resistant cells compared with TMZ-sensitive cells and NHAs (Fig. 4j). In order to further explore the mechanism of methylated changes in the promoter region of SNHG12, we detected the expression level of DNA methyltransferases (DNMTs) in TMZ-resistant cells. As is shown in Fig. 4k, DNMT1 expression was downregulated in TMZ-resistant cells compared with the parental TMZ-sensitive cells. Overexpression of DNMT1 led to increased methylation level in SNHG12 promoter region (Fig. 4l). Moreover, overexpression of DNMT1 significantly suppressed SNHG12 expression in TMZ-resistant cells,and knockdown of DNMT1 resulted in restoration of SNHG12 expression in TMZ-sensitive cells (Fig. 4m). ChIP assays revealed that the enrichment of SP1 at Site 3 of SNHG12 was decreased after DNMT1 overexpression, and knockdown of DNMT1 increased SP1 enrichment (Fig. 4n). These results suggest that DNMT1 was involved in epigenetic regulation process in SNHG12 promoter.

Several studies have shown that a high percentage of hypermethylated genes are pre-marked with H3K27me3 modifications, while hypomethylated genes show H3K4me3 modifications [20, 21]. Here, compared to TMZ-sensitive cells and NHAs, H3K27me3 was markedly decreased in TMZ-resistant cells, whereas H3K4me3 was significantly increased in TMZ-resistant cells (Additional file 8: Figure S3f). Thus, these data indicated that loss of DNA methylation makes the promoter region of SNHG12 more accessible to SP1, which leads to transcriptional activation of SNHG12.

### SNHG12 serves as a sponge for miR-129-5p

**Fig. 5:**
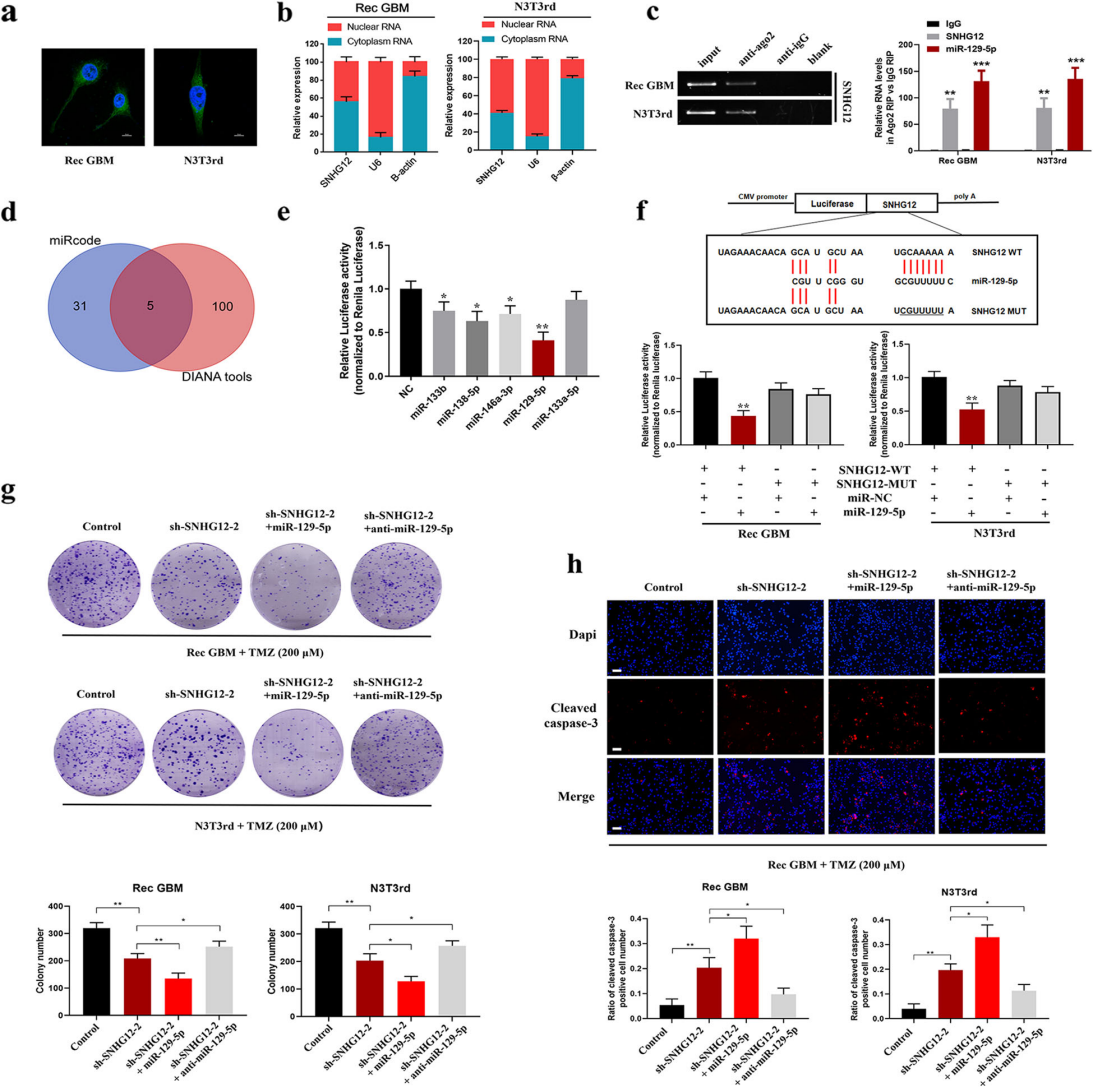
SNHG12 act as a sponge for miR-129-5p in the cytoplasm. **a** FISH analysis indicated subcellular location of SNHG12 in Rec GBM and N3T3rd cells (green). Nuclei were stained by DAPI (blue). **b** Relative SNHG12 expression levels in nuclear and cytosolic fractions of Rec GBM and N3T3rd cells. U6 was used as nuclear controls. β-actin was used as cytosolic controls. **c** RIP experiments were performed using the Ago2 antibody, and specific primers were used to detect the enrichment of SNHG12 and miR-129-5p in Rec GBM and N3T3rd cells. **d** Schematic drawing of the screening procedure of candidate miRNAs. **e** The luciferase reporter plasmids carrying SNHG12 was co-transfected into HEK293T cells with 5 miRNA-coding plasmids. **f** Up: Schematic representation of the miR-129-5p binding sites in SNHG12 and the site mutagenesis. Down: The luciferase reporter plasmid carrying wild type (WT) or mutant (MUT) SNHG12 was co-transfected into Rec GBM and N3T3rd cells with miR-129-5p in parallel with an empty vector. Relative luciferase activity in Rec GBM and N3T3rd cells were determined. **g** Colony formation ability of Rec GBM and N3T3rd cells transfected with SNHG12 plasmid, miR-129-5p mimics + SNHG12 plasmid or miR-129-5p inhibitor + SNHG12 plasmid after 200 μM TMZ treatment for 48 h. **h** Immunofluorescent staining of cleaved caspase-3 in Rec GBM cells transfected with SNHG12 plasmid, miR-129-5p mimics + SNHG12 plasmid or miR-129-5p inhibitor + SNHG12 plasmid after 200 μM TMZ treatment for 48 h. Scale bar = 50 μm. Data are presented as the mean ± SEM from three independent experiments. Significant results were presented as NS non-significant, *P<0.05, **P<0.01

Besides mediating epigenetic regulation in the nucleus, lncRNAs also regulate target gene expression by acting as competing endogenous RNAs (ceRNAs) for miRNAs in the cytoplasm. To investigate the molecular mechanisms underpinning the role of SNHG12 in acquired TMZ resistance in GBM, we firstly analyzed its distribution with the use of a nuclear mass separation assay and FISH analysis. SNHG12 was found to be a cytoplasm-enriched lncRNA (Fig. 5a and b). RNA-binding protein immunoprecipitation (RIP) indicated that SNHG12 and miR-129-5p binds directly with Argonaute-2 (Ago2), which is a core component of the RNA-induced silencing complex (RISC) involved in the miRNA-mediated repression of mRNAs (Fig. 5c). By using the miRcode and DIANA databases, five candidate miRNAs (miR-133b, miR-138-5p, miR-146a-3p, miR-129-5p, and miR-133a-5p) were predicted to have binding sites along the SNHG12 sequence (Fig. 5d). Next, dual luciferase reporter assays were used to confirm these prediction results. For this purpose, luciferase plasmids harboring the SNHG12 sequence together with plasmids encoding the individual miRNAs or the control sequence were transfected into HEK293T cells. The results showed that SNHG12-driven luciferase activities were suppressed by miR133b, miR-138-5p, miR-146a-3p, and miR-129-5p, with miR-129-5p showing the strongest suppression (Fig. 5e). Furthermore, miR-129-5p-mediated luciferase activity suppression was restored by mutation of SNHG12 within the predicted binding site for this miRNA (Fig. 5f). The expression level of miR-129-5p was found to be significantly decreased in GBM tissues based on analysis of CGGA miRNA expression microarray data (Additional file 9: Figure S4a). Moreover, the expression of miR-129-5p was down-regulated in TMZ-resistant cells compared with parental TMZ-sensitive cells, and knockdown of SNHG12 notably increased miR-129-5p expression levels in TMZ-resistant cells (Additional file 9: Figure S4b-c).

To investigate whether miR-129-5p participates in the SNHG12-mediated mechanism involved in acquired TMZ resistance in GBM, we knocked down or overexpressed miR-129-5p in SNHG12-depleted TMZ-resistant GBM cells. Colony formation assays showed that miR-129-5p overexpression markedly suppressed TMZ resistance in SNHG12-depleted TMZ-resistant cells, while knockdown of miR-129-5p reversed SNHG12 knockdown-mediated suppression of tumor cell proliferation and chemoresistance (Fig. 5g). Similarly, miR-129-5p overexpression increased TMZ-induced apoptosis in SNHG12-depleted TMZ-resistant cells, while miR-129-5p knockdown reversed SNHG12 knockdown-mediated apoptosis in TMZ-resistant cells (Fig. 5h and Additional file 9: Figure S4d-e). Together, these data suggested that SNHG12 functions as a molecular sponge for miR-129-5p, and both of these molecules are involved in the molecular mechanisms underlying acquired TMZ resistance in GBM.

### MAPK1 and E2F7 are miR-129-5p target genes and are responsible for SNHG12-mediated temozolomide resistance

**Fig. 6:**
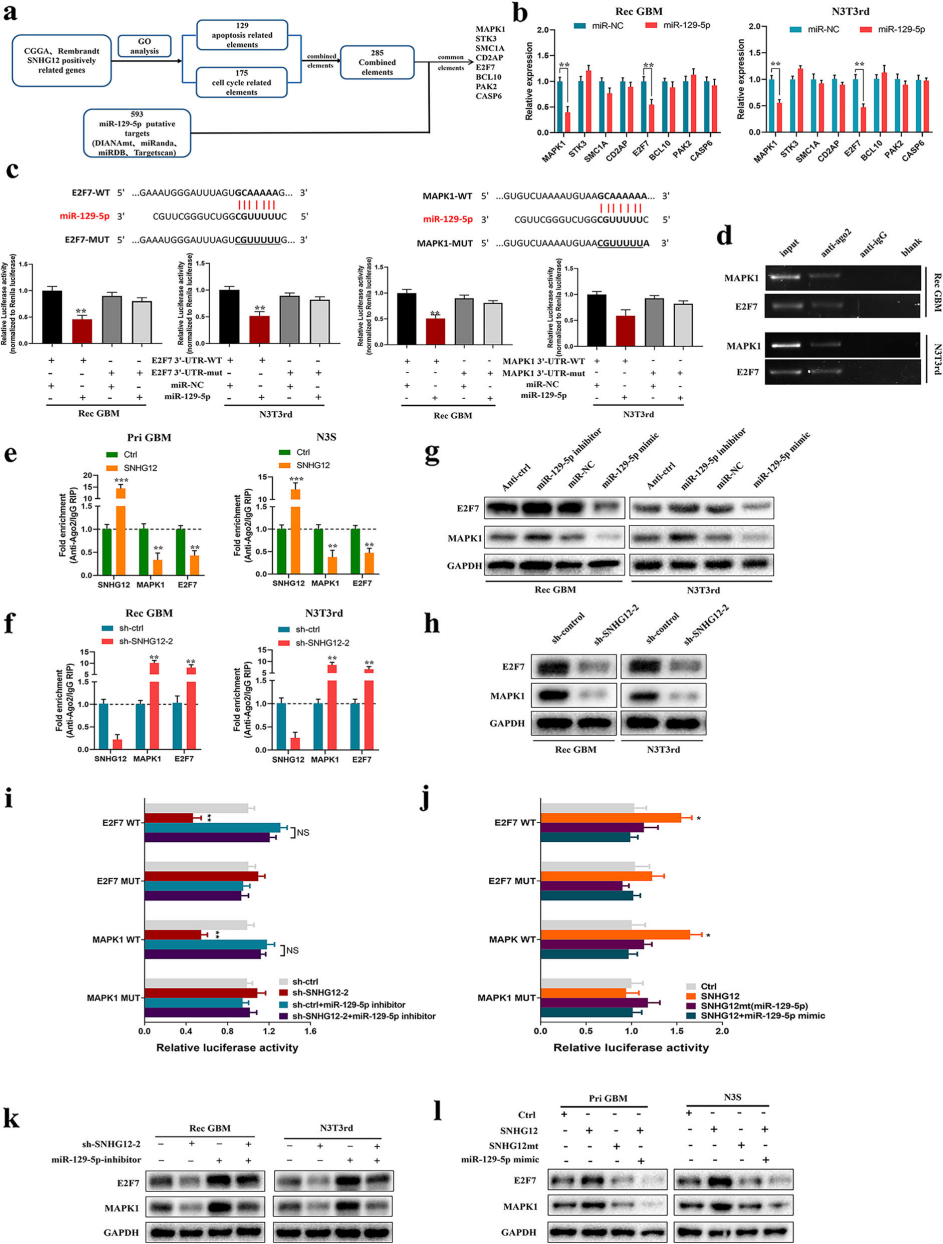
MAPK1 and E2F7 are direct targets of miR-129-5p and is suppressed by SNHG12 detection. **a** Schematic drawing of the screening procedure of candidate target genes. **b** After transfected with miR-NC or miR-129-5p in Rec GBM and N3T3rd cells, the expression level of 8 potential targets for miR-129-5p was analyzed using real-time PCR. **c** The luciferase reporter plasmid carrying wild type (WT) or mutant (MUT) MAPK1 or E2F7 was co-transfected into Rec GBM and N3T3rd cells with miR-129-5p in parallel with an empty vector. Relative luciferase activity in Rec GBM and N3T3rd cells were determined. **d** RIP experiments were performed using the Ago2 antibody, and specific primers were used to detect the enrichment of MAPK1 and E2F7. **e** RIP assay of the enrichment of Ago2 on SNHG12, MAPK1 and E2F7 transcripts relative to IgG in Pri GBM and N3S cells transfected with pcDNA-ctrl or pcDNA-SNHG12. **f** RIP assay of the enrichment of Ago2 on SNHG12, MAPK1 and E2F7 transcripts relative to IgG in Rec GBM and N3T3rd cells transfected sh-ctrl or sh-SNHG12. **g** Relative protein levels of E2F7 and MAPK1 in Rec GBM and N3T3rd cells transfected with control miRNA, miR-129-5p-inhibitor or miR-129-5p mimics. **h** MAPK1 and E2F7 protein level in Rec GBM and N3T3rd cells following knockdown of SNHG12. **i** and **j** Luciferase activity of reporters which contained wild-type or mt MAPK1 or E2F7 3’UTR with indicated treatment in Rec GBM cells (**i**) and Pri GBM cells (**j**) **k** Relative protein levels of E2F7 and MAPK1 in Rec GBM and N3T3rd cells following knockdown of SNHG12 and/or inhibition of miR-129-5p. **l** Western blot analysis of E2F7 and MAPK1 in Pri GBM cells and N3S cells transfected with pcDNA-Ctrl, pcDNA-SNHG12, or pcDNA-SNHG12mt along with miR-129-5p mimics. Data are presented as the mean ± SEM from three independent experiments. Significant results were presented as NS non-significant, **P<0.01, ***P<0.001

To identify the target genes of the ceRNA network between SNHG12 and miR-129-5p, four databases (DIANA, miRanda, miRDB, and TargetScan) were used to predict miR-129-5p targets, resulting in the identification of 593 putative targets. To increase the accuracy of these predictions, 285 candidate genes of which altered expression could contribute to aberrant cell cycle or apoptosis phenotypes were selected from among the SNHG12-associated genes in the CGGA and Rembrandt databases. Among these putative targets, we selected eight genes as potential components of the SNHG12 and miR-129-5p ceRNA network (Fig. 6a). Next, qRT-PCR revealed that overexpression of miR-129-5p significantly inhibited MAPK1 and E2F7 expression in Rec GBM cells and N3T3rd cells, while anti-miR-129-5p exhibited the opposite effect (Fig. 6b and Additional file 10: Figure S5a). We then analyzed MAPK1 and E2F7 expression in GBM cells and 20 recurrent GBM tissues. The results showed that MAPK1 and E2F7 were highly expressed in TMZ-resistant cells, meanwhile, SNHG12 was highly expressed in TMZ-resistant cells and this had a positive correlation with MAPK1 and E2F7 expression (Additional file 10: Figure S5b-c). The same correlation was found when analyzing data from the CGGA and Rembrandt databases (Additional file 10: Figure S5d). Furthermore, dual luciferase reporter assays revealed that miR-129-5p bound directly to the MAPK1 and E2F7 3′-UTR regions (Fig. 6c). RIP assays on Ago2 were performed, which revealed that MAPK1 and E2F7 can bind to Ago2 (Fig. 6d). Overexpression of SNHG12 in TMZ-sensitive cells led to the increased enrichment of Ago2 transcripts bound to SNHG12 but decreased enrichment of MAPK1 and E2F7 transcripts (Fig. 6e). In parallel, SNHG12 knockdown in TMZ-resistant cells elicited a marked increase in the recruitment of Ago2 to MAPK1 and E2F7 transcripts (Fig. 6f). These results suggested that SNHG12 can compete with MAPK1 and E2F7 transcripts for the Ago2-based miRNA-induced expression complex. MAPK1 and E2F7 protein levels were significantly increased and decreased by miR-129-5p inhibition and overexpression, respectively (Fig. 6g). In addition, knockdown of SNHG12 remarkably reduced MAPK1 and E2F7 mRNA and protein levels in Rec GBM and N3T3rd cells (Fig.6h and Additional file 10: Figure S5e). We then studied whether SNHG12-mediated sequestration of miR-129-5p was responsible for the upregulation of MAPK1 and E2F7. The luciferase activity of MAPK1 and E2F7 reporters was decreased upon SNHG12 knockdown and was rescued by miR-129-5p sponge, while the luciferase activity of mt reporters was unchanged (Fig. 6i). Conversely, the luciferase activity of MAPK1 and E2F7 wild-type reporters but not the mt ones was elevated upon pcDNA-SNHG12 transfection, whereas miR-129-5p overexpression abolished this effect (Fig. 6j). These results were further confirmed at both the RNA and protein levels of MAPK1 and E2F7 (Fig. 6k-l and Additional file 10: Figure S5f-g). Collectively, these data demonstrated that SNHG12 functions as a molecular sponge for miR-129-5p to facilitate the expression of MAPK1 and E2F7.

### MAPK1 and E2F7 exhibit different functions in SNHG12-mediated temozolomide resistance

**Fig. 7:**
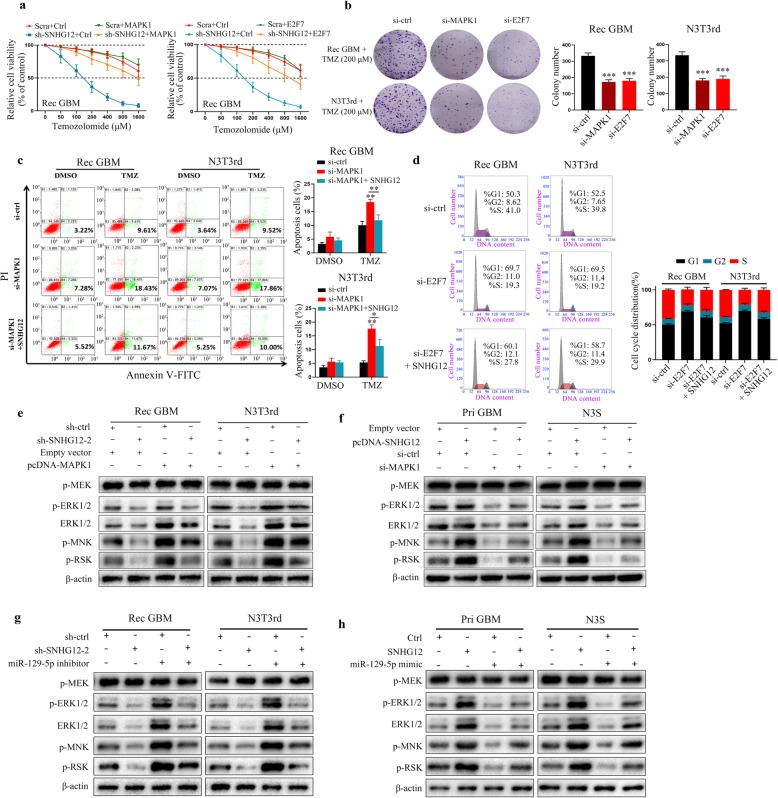
The effects of MAPK1 and E2F7 on GBM cell proliferation and survival. **a** CCK-8 assay analysis of the effect of MAPK1 or E2F7 overexpression on Rec GBM cells after knocking down SNHG12 upon TMZ treatment at the indicated concentrations for 48 h. **b** Colony formation analysis of Rec GBM and N3T3rd cells after co-transfected with si-ctrl, si-MAPK1 or si-E2F7. **c** Flow cytometric analysis of TMZ-induced (200 μM, 48 h) apoptosis in Rec GBM and N3T3rd cells transfected with si-ctrl or si-MAPK1. **d** The cell cycle distribution was analyzed by a flow cytometer in Rec GBM and N3T3rd cells. **e** Transfection of sh-SNHG12 or pcDNA-MAPK1 into Rec GBM and N3T3rd cells affected expression of MAPK signaling pathway-associated protein. Expression of the indicated proteins was detected by western blot. **f** Rescue assays for immunoblotting analysis of MAPK signaling pathway-associated protein after transfection of pcDNA-SNHG12 or si-MAPK1 into Pri GBM and N3S cells. **g** Western blot analysis of indicated proteins in Rec GBM cells and N3T3rd cells transfected with sh-SNHG12–2 and miR-129-5p inhibitor. **h** Western blot analysis of indicated proteins in Pri GBM cells and N3S cells transfected with pcDNA-SNHG12 and miR-129-5p mimics. Data are presented as the mean ± SEM from three independent experiments. Significant results were presented as NS non-significant, **P<0.01, ***P<0.001

After identifying MAPK1 and E2F7 as direct targets of SNHG12, we next analyzed the functional roles of MAPK1 and E2F7 in temozolomide resistance. E2F7 was previously reported to be a negative regulator of p21 transcription, leading to cyclin D1 up-regulation and cell cycle arrest [22, 23]. However, there is increasing evidence that E2F7 can promote cancer cell proliferation including in glioma [24, 25]. We observed that overexpression of MAPK1 and E2F7 restored the resistant phenotype in TMZ-resistant GBM cells with knocked-down SNHG12 (Fig. 7a and Additional file 11: Figure S6a). Next, we silenced MAPK1 and E2F7 with specific siRNAs in TMZ-resistant GBM cells (Additional file 11: Figure S6b). We found out that knockdown of MAPK1 or E2F7 in TMZ-resistant GBM cells markedly repressed cell proliferation and increased their sensitivity toward temozolomide (Fig. 7b). Interestingly, we observed that E2F7 dysregulation was primarily associated with G1/S cell cycle transition, while MAPK1 regulated both temozolomide-induced cell apoptosis and G1/S cell cycle transition and these effects can be abolished by SNHG12 overexpression (Fig. 7c, d and Additional file 11: Figure S6c-d). Further, we found that SNHG12-related genes in the CGGA and Rembrandt databases are associated with MAPK signaling pathway by using KEGG pathway analysis (Additional file 11: Figure S6e). MAPK1, also known as ERK2, is a component of the MAPK/ERK signaling pathway. In TMZ-resistant GBM cells with silenced SNHG12, we found that the total protein levels of ERK2 and phosphorylated ERK2 were reduced. Moreover, silencing of SNHG12 inhibited the phosphorylation of the downstream factors, MNK and RSK. All of these phenotypes were rescued by MAPK1 over-expression. In contrast, SNHG12 over-expression activated the MAPK/ERK pathway and rescued MAPK1 silencing in TMZ-resistant GBM cells (Fig. 7e-f and Additional file 11: Figure S6f). Moreover, inhibition of miR-129-5p was sufficient to restore downstream signaling following SNHG12 knockdown (Fig. 7g). Conversely, the introduction of miR-129-5p mimics abolished SNHG12-mediated downstream signaling (Fig. 7h). Thus, all these results demonstrated that MAPK1 and E2F7 exert different functions in SNHG12-mediated temozolomide resistance and that SNHG12 can regulate apoptosis through the MAPK/ERK pathway.

### Knockdown of SNHG12 restores TMZ sensitivity in vivo

**Fig. 8:**
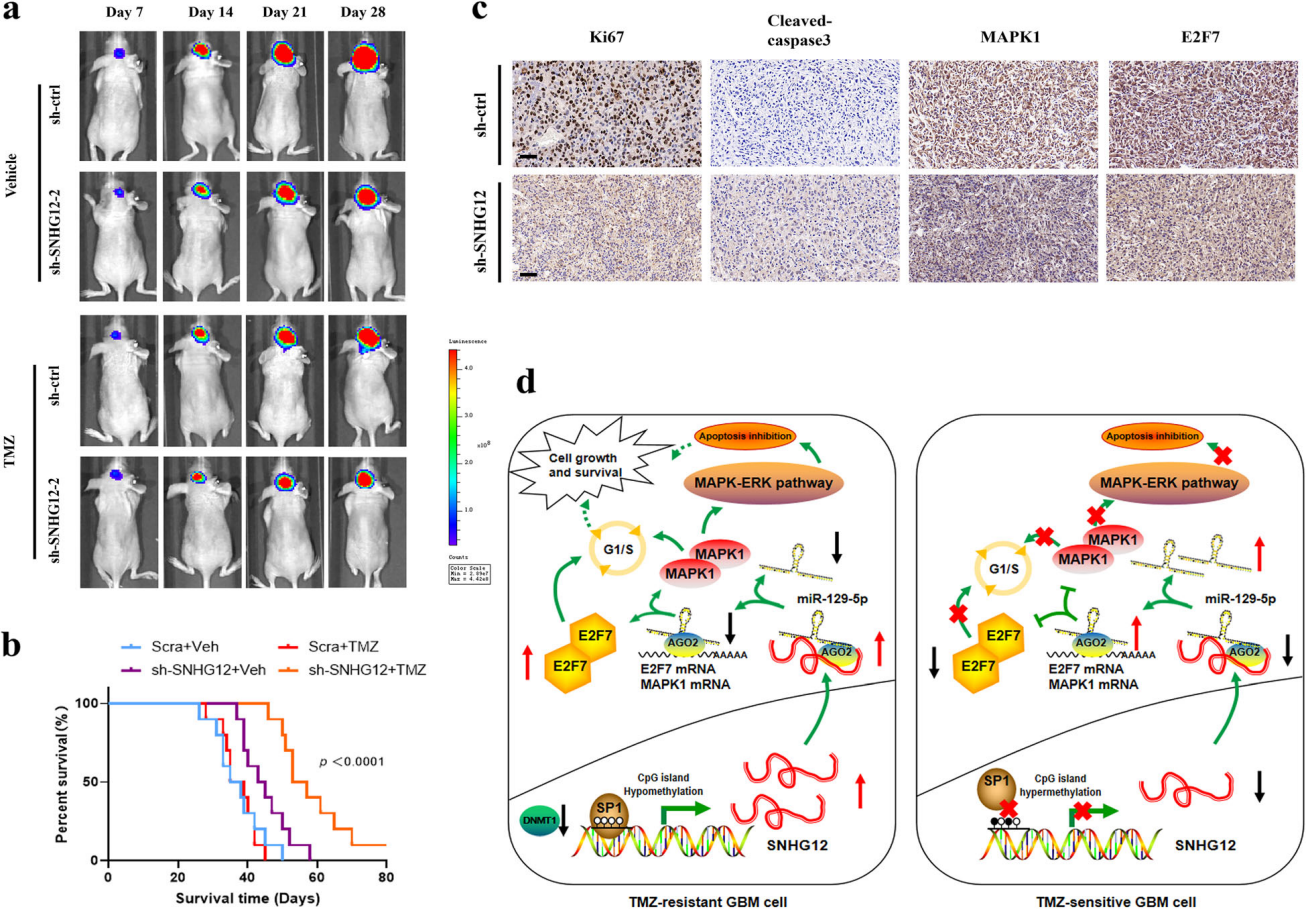
Knockdown of SNHG12 restores TMZ sensitivity in TMZ-resistant GBM xenografts. **a** Representative bioluminescence images of intracranial xenografts bearing SNHG12-depleted or control Rec GBM cells in the absence or presence of TMZ treatment on the days as indicated. **b** Immunohistochemistry staining of Ki67, Cleaved caspase-3, MAPK1 and E2F7. Scale bar = 50 μm (**c**) Kaplan-Meier survival curve of nude mice is shown. **d** The mechanistic scheme of DNA-methylation-mediated activating of lncRNA SNHG12 in regulating GBM cell proliferation and temozolomide resistance

To investigate the effect of SNHG12 on the TMZ-resistant phenotype in vivo, 2.5 × 10^5^ luciferase-labeled SNHG12-depleted or sh-ctrl-transduced recurrent GBM cells were injected into nude mice. Tumor progression was traced by in vivo bioluminescence imaging. The control xenografts showed tumor progression. In contrast, xenografts carrying SNHG12-depleted recurrent GBM cells displayed a significant regression in tumor growth. Next, the tumor-bearing mice were treated with TMZ (66 mg/kg/day, 5 days per week for three cycles) and analyzed by bioluminescence imaging. This revealed that SNHG12 depletion effectively restored the sensitivity of TMZ-resistant xenografts to TMZ treatment, and mice receiving this combined treatment had a prolonged lifespan (Fig. 8a–b). As shown by immunohistochemistry (IHC), the expression of Ki-67, MAPK1, and E2F7 decreased and the level of cleaved caspase-3 increased after knockdown of SNHG12 (Fig. 8c).

## Discussion

Surgical resection with postoperative radiotherapy and chemotherapy is currently the standard treatment for glioma patients. Clinical practice has proven that effective chemotherapy after successful surgery is one of the most effective methods for treating malignant glioma, and chemotherapy can significantly improve the survival rate and survival time of patients with malignant glioma. However, acquired TMZ resistance limits the range of therapeutic options available to GBM patients, particularly recurrent GBM patients. Elucidating the molecular mechanisms of TMZ resistance would greatly assist the rational design of combination therapies blocking TMZ chemotherapy resistance.

In recent years, the roles of lncRNAs in cancer have been widely studied. Many reports have shown that lncRNAs participate in several cancer-associated processes including chemoresistance [26, 27], and abnormal expression of lncRNAs have been shown to be involved in the malignant process of glioma [28–30]. However, lncRNA-mediated TMZ-resistant mechanisms have rarely been studied. Here, we identified lncRNA SNHG12 to be aberrantly expressed in TMZ-resistant cells and tissues, resulting in the TMZ-resistant phenotype. SNHG12 was shown to promote TMZ resistance by promoting cell growth and inhibiting cell apoptosis. Knockdown of SNHG12 increased the sensitivity of TMZ-resistant cells toward TMZ, which indicates that SNHG12 may be a key node of treatment intervention as part of GBM therapy.

Similar to protein-coding genes, lncRNA expression is affected by gene dosage and promoter utilization, through factors such as copy-number alterations and epigenetic regulation [31–34]. DNA methylation was one of the first modes of epigenetic regulation to be discovered. The DNA methylation landscape of progressive glioblastoma has been shown to exhibit extensive spatial and temporal heterogeneity, and the analysis of genome-wide DNA methylation patterns has proven helpful for glioma classification and diagnosis [35, 36]. However, aberrant changes in the DNA methylation patterns of lncRNAs are seldom reported. To identify the mechanisms underlying the abnormally high expression of SNHG12, we analyzed the promoter region of SNHG12. We observed abnormally low levels of methylation within this region, resulting in increased SNHG12 expression.

Studies have shown that transcription factors can also regulate the transcription of lncRNAs [37, 38]. We, therefore, investigated whether transcription factors play a role in the aberrant expression of SNHG12 and whether loss of DNA methylation would make the promoter region of SNHG12 more accessible to transcription factors. We found that SNHG12 is activated by the transcription factor, SP1. SP1 has been reported to play an important role in acquired TMZ resistance in GBM [39, 40], and our results revealed that SP1 activated the transcription of SNHG12. Moreover, loss of DNA methylation made the promoter of SNHG12 more accessible to this transcription factor. However, all the factors mediating the methylation of the SNHG12 promoter region have not yet been fully elucidated. Further study is needed to clarify these factors.

The molecular functions of lncRNAs are primarily determined by their subcellular localization [41]. Thus, to further unravel the downstream effects of SNHG12, we analyzed the localization of SNHG12 in TMZ-resistant cells. Our results showed that SNHG12 was expressed in both the nucleus and cytoplasm, with particularly high expression in the cytoplasm. LncRNAs have been shown to act as endogenous decoys for miRNAs, thereby forming complex regulatory networks [11, 42]. In this study, we determined that SNHG12 interacts with Ago2 in TMZ-resistant cells, which indicates that SNHG12 can act as a miRNA sponge. Further, by using bioinformatic analyses and luciferase reporter assays, we found miR-129-5p to be a potential target of SNHG12. Our previous study found that miR-129-5p overexpression could confer chemosensitivity to TMZ in GBM cells [43]. Taken together, these results indicate that the ceRNA network in which SNHG12 participates plays an important role in the mechanism of acquired TMZ resistance in glioma. Further, in the present study, we found that MAPK1 and E2F7 are potential targets of the SNHG12 ceRNA networks, which was validated by luciferase reporter assays and RIP analysis. MAPK1, as a member of the ERK signaling pathway, is associated with the mechanisms of chemoresistance in many cancers [44, 45]. The Ras/Raf/ERK (MAPK/ERK) pathway has been reported to be overactivated in many cancers, thereby promoting the malignant phenotype; it is, therefore, considered to be a potential drug target [46, 47]. E2F7, a member of the E2F family of transcription factors, is overexpressed in many cancers, which can induce cell proliferation in tumors such as glioma [25, 48]. In our study, we found that SNHG12 promotes TMZ resistance by competitively binding miR-129-5p, resulting in the dysregulation of MAPK1 and E2F7. We further validated that SNHG12-mediated G1/S cell cycle transitioning is mainly regulated by E2F7, while cell apoptosis is predominantly modulated by MAPK1. Furthermore, our results indicated that SNHG12 inhibit apoptosis by activating the MAPK/ERK pathway.

It is important to emphasize the potential limitations of this study. Firstly, our findings establish that SNHG12 axis is an important regulator of TMZ resistance. Thus, targeting this axis may present as a potential therapeutic strategy for treating GBM. However, Individual GBM are far from being uniform, and single tumors exhibit substantial cellular heterogeneity that includes small subpopulations termed glioma stem-like cells (GSCs). GSCs have been shown to contribute to tumor initiation, malignant phenotypes, recurrence and therapy-resistance. Drug resistance toward TMZ is multifactorial, involving not only the internal processes of cells but also factors within the GBM microenvironment [49, 50]. Secondly, analysis of recurrent glioma tissue demonstrated increased SNHG12 expression compared to primary, treatment naive tumors. Our previous study noted that serum ncRNAs were higher in GBM patients that responded poorly to TMZ. Given the ease of obtaining serum samples, analyzing serum SNHG12 level following TMZ treatment may be a potential strategy for predicting response to TMZ.

## Conclusions

Collectively, our results revealed that lncRNA SNHG12, which is activated by abnormal DNA demethylation and increased binding of transcription factor SP1, induces TMZ resistance in GBM. SNHG12 was found to play an important role in promoting cell proliferation and inhibiting cell apoptosis by acting as a sponge of miR-129-5p, thereby increasing MAPK1 and E2F7 expression and activating the MAPK-ERK pathway. Moreover, we identified that SNHG12 is correlated with poor overall survival and drug sensitivity in the clinic. Therefore, SNHG12 is a promising prognostic biomarker and a potential therapeutic target for temozolomide resistance in GBM.

## Supplementary information

**Additional file 1: Table S1.** Summary of clinical GBM patients.

**Additional file 2: Table S2.** Primer sequence used in this study and SNHG12 promoter sequence.

**Additional file 3: Table S3.** Information of antibodies.

**Additional file 4: Table S4.** Sequences of siRNA and shRNA against specific targets.

**Additional file 5: Table S5.** The differentially expressed lncRNAs in CGGA, Rembrandt and GEO data sets; **Table S6.** Predicted miRNAs targeting to SNHG12; **Table S7.** Predicted SNHG12 and miR-129-5p target genes.

**Additional file 6: Figure S1.** SNHG12 is up-regulated in GBM, related to Fig. 1.

**Additional file 7: Figure S2.** SNHG12 levels correlate with temozolomide resistance, related to Figs. 2-3.

**Additional file 8: Figure S3.** DNA methylation and SP1 regulate SNHG12 expression level, related to Fig. 4.

**Additional file 9: Figure S4.** SNHG12 act as a sponge for miR-129-5p in the cytoplasm, related to Fig. 5.

**Additional file 10: Figure S5.** SNHG12 regulates MAPK1 and E2F7 expression by competitively binding miR-129-5p, related to Fig. 6

**Additional file 11: Figure S6.** SNHG12 accelerates temozolomide resistance in GBM cells via MAPK1 and E2F7, related to Fig. 7.

## Data Availability

The datasets used and/or analyzed during the current study are available from the corresponding author on reasonable request.

## Abbreviations

Ago2: Argonaute 2
ANOVA: Analysis of variance
ceRNAs: Competing endogenous RNAs
CGGA: Chinese Glioma Genome Altas
ChIP: Chromatin immunoprecipitation
DMEM: Dulbecco’s modified Eagle’s medium
FISH: Fluorescence in situ hybridization
GBM: Glioblastoma multiforme
H3K27me3: Histone H3 lysine 27 trimethylation
H3K4me3: Histone H3 lysine 4 trimethylation
IHC: Immunochemistry
lncRNA: Long non-coding RNA
RIP: RNA immunopricipitation
SNHG12: Small nucleolar RNA host gene 12
SP1: Sp1 transcription factor
TMZ: Temozolomide
MAP K1: Mitogen-activated protein kinase 1
E2F7: E2F transcription factor 7
