# Supplementary material for: DNA-methylation-mediated activating of lncRNA SNHG12 promotes temozolomide resistance in glioblastoma
Source: Mol Cancer. 2020 Feb 10;19:28. doi: 10.1186/s12943-020-1137-5 (PMC7011291; doi:10.1186/s12943-020-1137-5)
Supplement: Supplementary file 1 — Additional file 1: Table S1. Summary of clinical GBM patients. [file 12943_2020_1137_MOESM1_ESM.docx]

**Additional file 1:**

**Table S1: Summary of clinical GBM patients**

| **Characteristic** | **All patient** | |
| --- | --- | --- |
|  | **Low**  **SNHG12 expression**  **(n=30)** | **High**  **SNHG12 expression**  **(n=30)** |
| **Type** |  |  |
| Pri GBM | 24 | 16 |
| Rec GBM | 6 | 14 |
| **Sex (n)** |  | |
| Male | 17 | 19 |
| Female | 13 | 11 |
| **age** |  |  |
| ≥45 | 26 | 24 |
| ＜45 | 4 | 6 |
| **Tumor location** |  | |
| Frontal | 12 | 14 |
| Non-frontal | 18 | 16 |
| **KPS score** |  |  |
| ≥80 | 24 | 19 |
| ＜80 | 6 | 11 |
| **MGMT promotor status** |  | |
| Methylated | 13 | 23 |
| Unmethylated | 17 | 7 |
| **Extent of surgery** |  |  |
| Total | 17 | 20 |
| Subtotal | 13 | 10 |
| **IDH1/2 genotype** |  | |
| Mutation | 5 | 4 |
| Wild-type | 25 | 26 |
| **TMZ dose** | 75 mg/m^2^/d after first surgery | |

**Abbreviations**: KPS, Karnofsky performance status; MGMT, O-6-methylguanine-DNA-methyltransferase; IDH1/2, isocitrate dehydrogenase 1 and 2; TMZ, temozolomide.
