# Supplementary material for: DNA-methylation-mediated activating of lncRNA SNHG12 promotes temozolomide resistance in glioblastoma
Source: Mol Cancer. 2020 Feb 10;19:28. doi: 10.1186/s12943-020-1137-5 (PMC7011291; doi:10.1186/s12943-020-1137-5)
Supplement: Supplementary file 2 — Additional file 2: Table S2. Primer sequence used in this study and SNHG12 promoter sequence. [file 12943_2020_1137_MOESM2_ESM.docx]

**Additional file 2: Table S2**

| **Sequences of primers used for qRT-PCR** | |
| --- | --- |
| **qPCR primer name** | **Sequence (5’-3’)** |
| SNGH12 (Forward) | GAAAAAGCACACCAGCTATTGG |
| SNGH12 (Reverse) | CGGGATCTCTGTAGACTAAGTCAGT |
| MALAT1 (Forward) | CTAAGGTCAAGAGAAGTGTCAG |
| MALAT1 (Reverse) | AAGACCTCGACACCATCGTTAC |
| CRNDE (Forward) | GGATGCTGTCAGCTAAGTTCAC |
| CRNDE (Reverse) | TTCCAGTGGCATCCTCCTTATC |
| MAPK1 (Forward) | AACAGGCTCTGGCCCACCCA |
| MAPK1 (Reverse) | AGTCCTCTGAGCCCTTGTCCTGA |
| E2F7 (Forward) | CTGCTGCGCTAGACTTGGAT |
| E2F7 (Reverse) | TCTCTTAGTAGGACCACCAACG |
| STK3 (Forward) | CCATCTGCCTTAGGAACGGA |
| STK3 (Reverse) | CGACAACTTGACCGGATTCC |
| SMC1A (Forward) | TACCTCAGTTCTCGGGCGTA |
| SMC1A (Reverse) | TGACTTACCAGAGCCATTGGG |
| CD2AP (Forward) | GGCATGGGAATGTAGCAAGTC |
| CD2AP (Reverse) | CCACCAGCCTTCTTCTACCTC |
| BCL10 (Forward) | TCATGTACCATCCAGAAG |
| BCL10 (Reverse) | AAGTGTAGTTGAAGAGAAGA |
| PAK2 (Forward) | ACCCTTTGTCAGCCAATCAC |
| PAK2 (Reverse) | AACATGGATGGTGTGCTCAA |
| CASP6 (Forward) | ACCTGCGCAGATAGAGACAA |
| CASP6 (Reverse) | CGTGGCTAACAGTTGACACC |
| miR-133b (Forward) | GCAGGTTTGGTCCCCTTCAAC |
| miR-133b (Reverse) | GTGCAGGGTCCGAGGT |
| miR-138-5p (Forward) | GCGAGCTGGTGTTGTGAATC |
| miR-138-5p (Reverse) | AGTGCAGGGTCCGAGGTATT |
| miR-146a-3p (Forward) | CGCGCCTCTGAAATTCAGTT |
| miR-146a-3p (Reverse) | AGTGCAGGGTCCGAGGTATT |
| miR-129-5p (Forward) | CGCTTTTTGCGGTCTGG |
| miR-129-5p (Reverse) | AGTGCAGGGTCCGAGGTATT |
| miR-133a-5p (Forward) | CGCGAGCTGGTAAAATGGAA |
| miR-133a-5p (Reverse) | AGTGCAGGGTCCGAGGTATT |
| U6 (Forward) | CTCGCTTCGGCAGCACA |
| U6 (Reverse) | AACGCTTCACGAATTTGCGT |
| GAPDH (Forward) | GAACGGGAAGCTCACTGG |
| GAPDH (Reverse) | GCCTGCTTCACCACCTTCT |
| β-actin (Forward) | GTCATTCCAAATATGAGATGCGT |
| β-actin (Reverse) | GCATTACATAATTTACACGAAAGCA |
| **Sequences of primers used for** **ChIP-PCR** | |
| **ChIP-PCR primer name** | **Sequence** **(5’-3’)** |
| SNHG12 Site 1 (Forward) | AAATTACATATTAGTGGAAGA |
| SNHG12 Site 1 (Reverse) | ATGTCTTTCAGTCTGGAA |
| SNHG12 Site 2 (Forward) | CAGGAAATCGAGACCATC |
| SNHG12 Site 2 (Reverse) | CAAGTAGCTGGGATTACA |
| SNHG12 Site 3 (Forward) | ACGACCTGCTAAAGTAGTAC |
| SNHG12 Site 3 (Reverse) | CAGGAGAATGGCGTGAAC |
| **Sequences of primers used for BSP** | |
| **BSP primer name** | **Sequence (5’-3’)** |
| SNHG12 BSP1 (Forward) | TAGGAAGTYGATTYGTTTTGG |
| SNHG12 BSP1 (Reverse) | CCTTTRGAAAAATATAACCCCC |
| SNHG12 BSP2 (Forward) | GAAGGTAGGTAGAGGTTTGTAGTTAG |
| SNHG12 BSP2 (Reverse) | CCAACACACAAAATTCATTTCC |
| **Sequences of primers used for MSP** | |
| **MSP primer name** | **Sequence (5’-3’)** |
| SNHG12 M1 (Forward) | GGGTTTACGTAGGAAGTCGAT |
| SNHG12 M1 (Reverse) | GAAAAATATAACCCCCTCCCC |
| SNHG12 U1 (Forward) | TGGGTTTATGTAGGAAGTTGA |
| SNHG12 U1 (Reverse) | AAAAAATATAACCCCCTCCCC |

**SNHG12 promoter sequence （CpG islands）**

>Homo sapiens:chr1:28907966:28908866:- （TSS: -500 to +400）

cacagttaaaatagcacgcgctcagtaaAATTACATATTAGTGGAAGAGACAAGCCTTGAATAAATCGCCTTTCGGGGAGTATGACCCCCTCCCCGAAACCCACTCAGGCGCTGCTCGCTCGCACGCCCACTGAGCTCGGAGCGGAGCCCGAGCCCTTTCCCAGCACACAGGGTTCATTTCCAGACTGAAAGACATCCCAGAGACAACTCCAGAGGCCAAGGCGGGTCGGCTTCCTGCGTGGGCCCAGCGCCGGGCACTGAAAGGTGAAGGCGCTTCTTCCGCTCGGAGGAAAGCCTCTGGCGGTTCTCGCTTCGCGGGCTCGGGATGGACACGCGCGTAGGGCTGGGGAGGTGACGCGCGGACAACGCCGGGGCGAGCAAACTACAACTCCCAGGCAGCTGTGCGCCACGCGGGCGGCCTACTGTCGGGTGTGGCCGCGAGGGGCGGCCTATATAAGCTCTTGCTCGCGGCTTCAGCGCCCTTTCTCCCCGCCGCATTCCCGGTGTCGACTTACTAGCTGCAAGCCTCTGCCTGCCTTCCTGCGCGCCGTTCCCCGCTAGTCGCTGCTGCTGGCGCGCACTCGCCGGGTTTTTCCTCCCACGGCCTCGAGATGGTGGTGAATGTGGCACGGAGGAGCCGGGCCTTCCAACCCGGTGGGCCCGAGCTCCGAAAGGCCCCCTCGGCAGTGAGAggggcgggagcccgcgggggccgcgcccTTCTCTCGCTTCGGACTGCGCAACGCTGCGCTCTGGGCTGACAGGTGAGTGTTGCAGGCAGGCGGCCGGGTTTACGGAAGGGGTGGGGGTTTGGGAACCGGTCTCCTGGGGGATGCGGGGATGAATCCCTGGGCCTGAAGTGTCTTAATTCAGGGGGAGGCCGCATGCTGGTGTCAGGAT
