# Supplementary material for: DNA-methylation-mediated activating of lncRNA SNHG12 promotes temozolomide resistance in glioblastoma
Source: Mol Cancer. 2020 Feb 10;19:28. doi: 10.1186/s12943-020-1137-5 (PMC7011291; doi:10.1186/s12943-020-1137-5)
Supplement: Supplementary file 3 — Additional file 3: Table S3. Information of antibodies. [file 12943_2020_1137_MOESM3_ESM.docx]

**Additional file 3: Table S3**

**Table S3: List of antibodies**

| **Antibody** | **Catalogue NO.** | **Company** |
| --- | --- | --- |
| Caspase-3 | 9662 | Cell Signaling Technology (Beverly, MA) |
| SP1 | 9389 | Cell Signaling Technology (Beverly, MA) |
| Rb | 9309 | Cell Signaling Technology (Beverly, MA) |
| p-Rb | 9307 | Cell Signaling Technology (Beverly, MA) |
| p-MEK1/2 | 9154 | Cell Signaling Technology (Beverly, MA) |
| ERK1/2 | 4695 | Cell Signaling Technology (Beverly, MA) |
| p-ERK1/2 | 4370 | Cell Signaling Technology (Beverly, MA) |
| p-MNK1 | 2111 | Cell Signaling Technology (Beverly, MA) |
| GAPDH | 5174 | Cell Signaling Technology (Beverly, MA) |
| β-actin | 3700 | Cell Signaling Technology (Beverly, MA) |
| CDK4 | sc-70831 | Santa Cruz Biotechnology (Santa Cruz, CA) |
| CDK6 | sc-7961 | Santa Cruz Biotechnology (Santa Cruz, CA) |
| PARP | sc-8007 | Santa Cruz Biotechnology (Santa Cruz, CA) |
| Cyclin D1 | sc-20044 | Santa Cruz Biotechnology (Santa Cruz, CA) |
| E2F7 | ab56022 | Abcam (Cambridge, UK) |
| ERK2 | ab32081 | Abcam (Cambridge, UK) |
| p-RSK2 | ab75820 | Abcam (Cambridge, UK) |
| Ago2 | ab32381 | Abcam (Cambridge, UK) |
| DNMT1 | ab134148 | Abcam (Cambridge, UK) |
| DNMT3a | ab232391 | Abcam (Cambridge, UK) |
| DNMT3b | ab239893 | Abcam (Cambridge, UK) |
