# Supplementary material for: DNA-methylation-mediated activating of lncRNA SNHG12 promotes temozolomide resistance in glioblastoma
Source: Mol Cancer. 2020 Feb 10;19:28. doi: 10.1186/s12943-020-1137-5 (PMC7011291; doi:10.1186/s12943-020-1137-5)
Supplement: Supplementary file 4 — Additional file 4: Table S4. Sequences of siRNA and shRNA against specific targets. [file 12943_2020_1137_MOESM4_ESM.docx]

**Additional file 4: Table S4**

**Table S4: Sequences of siRNA and shRNA against specific targets**

| **Sequences of siRNAs** | | |
| --- | --- | --- |
|  | **sense sequence (5’-3’)** | **anti-sense sequence (5’-3’)** |
| si-SP1 | CCAGCAACAUGGGAAUUAUdTdT | AUAAUUCCCAUGUUGCUGGdTdT |
| si-MAPK1 | CAUGGUAGUCACUAACAUAdTdT | UAUGUUAGUGACUACCAUGdAdT |
| si-E2F7 | GCACCAAAGAGCCUUCUUUdTdT | AAAGAAGGCUCUUUGGUGCdTdT |
| si-DNMT1 | CGAGUCUGGCUUUGAGAGUdTdT | ACUCUCAAAGCCAGACUCGdTdT |
| **Sequences of shRNAs** | | |
|  | **sense sequence (5’-3’)** | **anti-sense sequence (5’-3’)** |
| sh-SNHG1#1 | GCAGUGUGCUACUGAACUUTT | AAGUUCAGUAGCACACUGCTT |
| sh-SNHG1#2 | GCUACUGAACUUCAGAGGUTT | ACCUCUGAAGUUCAGUAGCTT |
| sh-SNHG1#3 | GCUGUCCUCAUUUGUGACUAUTT | AUAGUCACAAAUGAGGACAGCTT |
| **Sequences for has-miR-129-5p** | | |
|  | **sequence (5’-3’)** | |
| hsa-miR-129-5p mimics (sense) | CUUUUUGCGGUCUGGGCUUGC | |
| hsa-miR-129-5p mimics (anti-sense) | GCAAGCCCAGACCGCAAAAAG | |
| hsa-miR-129-5p inhibitor (anti-sense) | GCAAGCCCAGACCGCAAAAAG | |
| miR-NC (sense) | UUUGUACUACACAAAAGUACUG | |
| miR-NC (anti-sense) | CAGUACUUUUGUGUAGUACAAA | |
| Anti-NC (anti-sense) | CAGUACUUUUGUGUAGUACAAA | |
