# Supplementary material for: DNA-methylation-mediated activating of lncRNA SNHG12 promotes temozolomide resistance in glioblastoma
Source: Mol Cancer. 2020 Feb 10;19:28. doi: 10.1186/s12943-020-1137-5 (PMC7011291; doi:10.1186/s12943-020-1137-5)
Supplement: Supplementary file 5 — Additional file 5: Table S5. The differentially expressed lncRNAs in CGGA, Rembrandt and GEO data sets; Table S6. Predicted miRNAs targeting to SNHG12; Table S7. Predicted SNHG12 and miR-129-5p target genes. [file 12943_2020_1137_MOESM5_ESM.docx]

**Additional file 5:**

**Table S5: The differentially expressed lncRNAs in CGGA, Rembrandt and GEO data sets**

| **CGGA** | | | |
| --- | --- | --- | --- |
| **lncRNAs** | **Log_2_FC** | **P.Value** | **adj.P.Val** |
| CRNDE | 3.552650074 | 2.02E-05 | 0.000641233 |
| MALAT1 | 2.104828751 | 4.04E-05 | 0.001012865 |
| SNHG1 | 1.778161289 | 3.18E-06 | 0.000184866 |
| KCNQ1OT1 | 1.678489067 | 0.005148113 | 0.034348041 |
| FAM138A | 1.440256974 | 1.62E-07 | 2.98E-05 |
| SNHG12 | 1.122257897 | 1.78E-05 | 0.000588288 |
| MIR17HG | 1.121465886 | 0.001924052 | 0.016828526 |
| FAM41C | 1.035001054 | 7.51E-07 | 7.68E-05 |
| **Rembrandt** | | | |
| **lncRNAs** | **Log_2_FC** | **P.Value** | **adj.P.Val** |
| CRNDE | 3.759580615 | 3.21E-20 | 1.15E-18 |
| NEAT1 | 1.924762859 | 3.96E-11 | 3.23E-10 |
| H19 | 1.799363537 | 4.21E-05 | 0.000126343 |
| LOC102724441 | 1.71738292 | 4.14E-14 | 5.34E-13 |
| ANKRD10-IT1 | 1.555432782 | 3.43E-14 | 4.47E-13 |
| IPW | 1.449425616 | 3.62E-14 | 4.71E-13 |
| MALAT1 | 1.35899387 | 0.000733484 | 0.001731185 |
| PSMB8-AS1 | 1.32380885 | 1.40E-17 | 3.10E-16 |
| LOC101060604 | 1.307505173 | 1.86E-14 | 2.52E-13 |
| LOC728613 | 1.27414708 | 8.71E-08 | 4.18E-07 |
| SNHG12 | 1.222374982 | 4.85E-18 | 1.15E-16 |
| IGFBP7-AS1 | 1.161970002 | 3.56E-05 | 0.00010819 |
| LOC400043 | 1.151040566 | 8.78E-11 | 6.78E-10 |
| **GPL570-merged (GSE4290, GSE7696, GSE15824, GSE50161)** | | | |
| **lncRNAs** | **Log_2_FC** | **pfp** | **P.value** |
| CRNDE | 3.451846738 | 0 | 0 |
| LINC01279 | 1.96772924 | 0 | 0 |
| LOC100131541 | 1.860811314 | 0 | 0 |
| H19 | 1.697216565 | 0 | 0 |
| DLEU2 | 1.628664495 | 0 | 0 |
| HOTAIRM1 | 1.624959376 | 0 | 0 |
| ANKRD10-IT1 | 1.582528881 | 0 | 0 |
| LINC01004 | 1.554001554 | 0 | 0 |
| MALAT1 | 1.53233221 | 0 | 0 |
| GABPB1-AS1 | 1.499025633 | 0 | 0 |
| ZFAS1 | 1.465630954 | 1.00E-04 | 0 |
| TUG1 | 1.459002043 | 0 | 0 |
| SNHG12 | 1.455815985 | 0 | 0 |
| LINC01355 | 1.445504481 | 2.00E-04 | 0 |
| LINC00673 | 1.444460494 | 0 | 0 |
| LOC102606465 | 1.428775539 | 0 | 0 |
| LOC100272216 | 1.414027149 | 0 | 0 |
| HOTAIR | 1.379500621 | 0 | 0 |
| ZFHX4-AS1 | 1.362954886 | 0 | 0 |
| TPT1-AS1 | 1.353729525 | 0.0025 | 2.00E-04 |
| AGAP2-AS1 | 1.339764202 | 0 | 0 |
| PAXIP1-AS1 | 1.336898396 | 0 | 0 |
| HNRNPU-AS1 | 1.327668614 | 0.0077 | 8.00E-04 |
| PITPNA-AS1 | 1.325908247 | 0.0014 | 1.00E-04 |
| LOC400043 | 1.308386759 | 0 | 0 |
| MEG3 | 1.302931596 | 0 | 0 |
| PSMA3-AS1 | 1.298195508 | 0.0032 | 3.00E-04 |
| LOC102724356 | 1.295672454 | 0.001 | 1.00E-04 |
| LOC642361 | 1.292991983 | 0.001 | 1.00E-04 |
| FLJ32255 | 1.291822762 | 0 | 0 |
| LINC01116 | 1.282544568 | 0 | 0 |
| LINC00998 | 1.279099514 | 8.00E-04 | 1.00E-04 |
| LOC101927027 | 1.271294177 | 0 | 0 |
| RASSF8-AS1 | 1.257703434 | 0 | 0 |
| LINC01354 | 1.256123603 | 0.0079 | 8.00E-04 |
| LINC00342 | 1.254862593 | 8.00E-04 | 1.00E-04 |
| LINC00663 | 1.253761284 | 0.0021 | 2.00E-04 |
| LINC01560 | 1.251251251 | 0 | 0 |
| NFIA-AS2 | 1.238696891 | 0 | 0 |
| LINC00662 | 1.232893601 | 0.0063 | 6.00E-04 |
| ADAMTS9-AS2 | 1.230314961 | 0.0045 | 4.00E-04 |
| LOC729218 | 1.225640397 | 0.0011 | 1.00E-04 |
| LINC01158 | 1.222942399 | 0 | 0 |
| TMEM161B-AS1 | 1.218026797 | 0 | 0 |
| LOC100129034 | 1.217730151 | 0 | 0 |
| RNASEH1-AS1 | 1.21743365 | 0.0066 | 7.00E-04 |
| LOC100131564 | 1.214181642 | 0.0037 | 4.00E-04 |
| PSMB8-AS1 | 1.207583625 | 0 | 0 |
| LOC101928847 | 1.206418145 | 0.0034 | 3.00E-04 |
| PAX8-AS1 | 1.201923077 | 0 | 0 |
| RMST | 1.198178768 | 0 | 0 |
| SPRY4-IT1 | 1.197174668 | 0 | 0 |
| NNT-AS1 | 1.196888091 | 0.0081 | 9.00E-04 |
| ZEB1-AS1 | 1.195028681 | 0.0082 | 9.00E-04 |
| UBL7-AS1 | 1.19431506 | 0.0041 | 4.00E-04 |
| LINC00869 | 1.194172439 | 3.00E-04 | 0 |
| RNF157-AS1 | 1.191327138 | 0 | 0 |
| ZNF790-AS1 | 1.187366421 | 0.0058 | 6.00E-04 |
| LOC101927365 | 1.185114956 | 0.0022 | 2.00E-04 |
| LINC01003 | 1.175502527 | 0 | 0 |
| LOC100507412 | 1.173984503 | 4.00E-04 | 0 |
| LINC01114 | 1.163331782 | 3.00E-04 | 0 |
| LOC646903 | 1.162115049 | 0.0025 | 2.00E-04 |
| XIST | 1.157273464 | 0 | 0 |
| SOX21-AS1 | 1.149161112 | 1.00E-04 | 0 |
| ARHGEF26-AS1 | 1.143118427 | 0 | 0 |
| LOC730101 | 1.141943588 | 9.00E-04 | 1.00E-04 |
| TP73-AS1 | 1.140771161 | 3.00E-04 | 0 |
| LINC01088 | 1.139341461 | 0 | 0 |
| TAPT1-AS1 | 1.138433515 | 0.0048 | 5.00E-04 |
| LOC645321 | 1.137009665 | 0 | 0 |
| DANCR | 1.135847342 | 0.0035 | 3.00E-04 |
| LOC729970 | 1.13237459 | 0.0078 | 8.00E-04 |
| LOC100134822 | 1.131221719 | 0.0089 | 0.001 |
| GBAT2 | 1.128795575 | 9.00E-04 | 1.00E-04 |
| LOC101927204 | 1.128540797 | 4.00E-04 | 0 |
| HCP5 | 1.118943717 | 1.00E-04 | 0 |
| LINC01268 | 1.116694584 | 0 | 0 |
| LOC100506844 | 1.107787748 | 0.0017 | 1.00E-04 |
| PAXIP1-AS2 | 1.105216622 | 0.0048 | 5.00E-04 |
| CARD8-AS1 | 1.101200308 | 0.0086 | 9.00E-04 |
| LINC01351 | 1.095650268 | 0 | 0 |
| DUXAP10 | 1.092896175 | 0 | 0 |
| LINC00623 | 1.091941472 | 0.0043 | 4.00E-04 |
| LOC100506123 | 1.090869423 | 0.0025 | 2.00E-04 |
| SOX9-AS1 | 1.089087345 | 0.0033 | 3.00E-04 |
| KANSL1-AS1 | 1.083775875 | 0 | 0 |
| LINC00645 | 1.078865034 | 0.0081 | 9.00E-04 |
| LINC01094 | 1.078515962 | 0 | 0 |
| LOC101929787 | 1.077586207 | 0.0064 | 7.00E-04 |
| LINC01198 | 1.058201058 | 0.0035 | 3.00E-04 |
| LINC01410 | 1.050751287 | 0.0076 | 8.00E-04 |
| LOC285097 | 1.035411058 | 0 | 0 |
| TTTY15 | 1.008878128 | 0 | 0 |
| LOC102723678 | 1.004217714 | 5.00E-04 | 0 |

**Table S6: Predicted miRNAs targeting to SNHG12**

| miRcode  32 miRNAs targeting SNHG12 | DIANA tools  105 miRNAs targeting SNHG12 |
| --- | --- |
| hsa-miR-503 | hsa-miR-6739-5p |
| hsa-miR-133b | hsa-miR-6733-5p |
| hsa-miR-9/9ab | hsa-miR-409-3p |
| hsa-miR-138-5p | hsa-miR-4683 |
| hsa-miR-140/140-5p/876-3p/1244 | hsa-miR-4775 |
| hsa-miR-146a-3p | hsa-miR-6773-3p |
| hsa-miR-148ab-3p/152 | hsa-miR-5193 |
| hsa-miR-150/5127 | hsa-miR-7977 |
| hsa-miR-15abc/16/16abc/195/322/424/497/1907 | hsa-miR-4684-3p |
| hsa-miR-181abcd/4262 | hsa-miR-129-5p |
| hsa-let-7/98/4458/4500 | hsa-miR-215-3p |
| hsa-miR-187 | hsa-miR-5003-3p |
| hsa-miR-193/193b/193a-3p | hsa-miR-1237-3p |
| hsa-miR-194 | hsa-miR-330-5p |
| hsa-miR-199ab-5p | hsa-miR-379-3p |
| hsa-miR-1ab/206/613 | hsa-miR-4674 |
| hsa-miR-200bc/429/548a | hsa-miR-668-3p |
| hsa-miR-204/204b/211 | hsa-miR-1273g-3p |
| hsa-miR-208ab/208ab-3p | hsa-miR-4659b-3p |
| hsa-miR-217 | hsa-miR-3680-3p |
| hsa-miR-218/218a | hsa-miR-4261 |
| hsa-miR-219-5p/508/508-3p/4782-3p | hsa-miR-3158-5p |
| hsa-miR-122/122a/1352 | hsa-miR-4448 |
| hsa-miR-24/24ab/24-3p | hsa-miR-326 |
| hsa-miR-338/338-3p | hsa-miR-494-3p |
| hsa-miR-33ab/33-5p | hsa-miR-935 |
| hsa-miR-425/425-5p/489 | hsa-miR-590-3p |
| hsa-miR-10abc/10a-5p | hsa-miR-1184 |
| hsa-miR-451 | hsa-miR-4496 |
| hsa-miR-129-5p | hsa-miR-4659a-3p |
| hsa-miR-499-5p | hsa-miR-3120-5p |
| hsa-miR-133a-5p | hsa-miR-1288-5p |
|  | hsa-miR-411-3p |
|  | hsa-miR-548f-3p |
|  | hsa-miR-6807-3p |
|  | hsa-miR-6732-3p |
|  | hsa-miR-515-5p |
|  | hsa-miR-5100 |
|  | hsa-miR-4691-3p |
|  | hsa-miR-4797-3p |
|  | hsa-miR-6875-3p |
|  | hsa-miR-495-3p |
|  | hsa-miR-670-3p |
|  | hsa-miR-133a-5p |
|  | hsa-miR-646 |
|  | hsa-miR-138-5p |
|  | hsa-miR-4474-5p |
|  | hsa-miR-5004-5p |
|  | hsa-miR-519e-5p |
|  | hsa-miR-29c-5p |
|  | hsa-miR-4660 |
|  | hsa-miR-3153 |
|  | hsa-miR-1247-5p |
|  | hsa-miR-4437 |
|  | hsa-miR-7114-5p |
|  | hsa-miR-1914-5p |
|  | hsa-miR-1285-3p |
|  | hsa-miR-3613-3p |
|  | hsa-miR-4641 |
|  | hsa-miR-133a-3p |
|  | hsa-miR-4728-3p |
|  | hsa-miR-5691 |
|  | hsa-miR-6856-3p |
|  | hsa-miR-1301-3p |
|  | hsa-miR-548as-3p |
|  | hsa-miR-146a-3p |
|  | hsa-miR-620 |
|  | hsa-miR-133b |
|  | hsa-miR-4778-3p |
|  | hsa-miR-5047 |
|  | hsa-miR-548g-3p |
|  | hsa-miR-548z |
|  | hsa-miR-548h-3p |
|  | hsa-miR-541-3p |
|  | hsa-miR-4766-5p |
|  | hsa-miR-589-3p |
|  | hsa-miR-4485-5p |
|  | hsa-miR-1226-3p |
|  | hsa-miR-4698 |
|  | hsa-miR-6884-5p |
|  | hsa-miR-6734-3p |
|  | hsa-miR-4670-3p |
|  | hsa-miR-3202 |
|  | hsa-miR-4524a-3p |
|  | hsa-miR-6071 |
|  | hsa-miR-6764-5p |
|  | hsa-miR-502-5p |
|  | hsa-miR-222-5p |
|  | hsa-miR-4314 |
|  | hsa-miR-548ac |
|  | hsa-miR-6765-3p |
|  | hsa-miR-6889-3p |
|  | hsa-miR-6776-5p |
|  | hsa-miR-3942-3p |
|  | hsa-miR-924 |
|  | hsa-miR-3120-3p |
|  | hsa-miR-6760-3p |
|  | hsa-miR-1270 |
|  | hsa-miR-197-3p |
|  | hsa-miR-1260b |
|  | hsa-miR-3678-3p |
|  | hsa-miR-6830-5p |
|  | hsa-miR-3622b-3p |
|  | hsa-miR-548az-3p |
|  | hsa-miR-6810-3p |

**Table S7: Predicted SNHG12 and miR-129-5p target genes**

| CGGA, Rembrandt  SNHG12 positively related genes | | miR-129-5p putative targets  (DIANAmt, miRanda, miRDB, Targetscan) |
| --- | --- | --- |
| Apoptosis  related elements | Cell cycle  related elements | ZBTB33 |
|  |  | PIGK |
| ITGB3BP | ITGB3BP | DNAJB6 |
| MCL1 | E2F2 | ABCC5 |
| MKNK2 | ZAK | ATP9A |
| CHEK2 | DBF4 | ARPC5 |
| DIDO1 | E2F8 | TSHZ1 |
| CASP6 | KNTC1 | UBE4B |
| CDCA7 | AURKA | CDKN1C |
| NOD1 | PTTG1 | CACNG2 |
| BUB1 | CDT1 | SEMA3A |
| DYRK2 | KIF2C | DLC1 |
| CASP2 | MCM8 | SEC23A |
| BCL10 | CDCA8 | CIB2 |
| AIMP1 | CDCA7 | SLU7 |
| PKN2 | SEH1L | SORBS1 |
| BIRC5 | CDCA2 | CDC42EP3 |
| IFI16 | RALA | MXD4 |
| BCL2L12 | NUP37 | EXOC5 |
| DRAM2 | ASPM | CUGBP2 |
| PDCL3 | CDCA3 | CSPG5 |
| KIF1B | ESCO1 | PLK4 |
| RIPK2 | CDC6 | NUP50 |
| PDCD6IP | KIF11 | HBS1L |
| PDCD5 | ZC3HC1 | NPFFR2 |
| APBB1 | ANAPC4 | MAN1A2 |
| MELK | PKN2 | IFI44L |
| DAP3 | HMG20B | B3GNT1 |
| S100A8 | MCM3 | KRR1 |
| S100A9 | UBE2C | PTPRT |
| RBM5 | TACC3 | KIF3A |
| RNF216 | ESCO2 | LDB3 |
| SMNDC1 | WEE1 | CHML |
| CTNNBL1 | RBBP8 | DUSP10 |
| CLPTM1L | UHRF1 | C14orf126 |
| NLRC4 | RCC2 | KLF12 |
| C8ORF4 | SPAG5 | GLCCI1 |
| DAP | ZWINT | CHRNA3 |
| CIB1 | SDCCAG3 | TRIM9 |
| SGPL1 | PDCD6IP | SLC25A25 |
| BCL2A1 | HAUS8 | KLHL32 |
| PIM1 | MELK | TMEM123 |
| FADD | TIPIN | GBP4 |
| STK4 | CHEK1 | FCHO2 |
| STK3 | CHEK2 | C20orf108 |
| MAPK1 | CEP55 | MIST |
| TNFAIP8 | RCC1 | ADD3 |
| TNFAIP3 | CENPA | LRIG3 |
| NEK6 | NCAPG | C14orf28 |
| ZFAND6 | USP39 | CCR1 |
| LITAF | BUB1 | CCDC43 |
| TMEM214 | SKA2 | CNN3 |
| ADAMTSL4 | ERCC6L | KLF17 |
| TMEM219 | PRPF40A | COL5A1 |
| STK17A | TAF1 | ANKRD54 |
| PTRH2 | PDS5A | COL19A1 |
| DDX47 | DLGAP5 | ADAT2 |
| UBE2D3 | NASP | COX15 |
| TRAF7 | SMC5 | ADHFE1 |
| TRAF5 | NUF2 | MUM1L1 |
| SRGN | CENPF | SPIN4 |
| CFLAR | KIF18B | RNF32 |
| CARD8 | NDC80 | MPP7 |
| TGFBR1 | BIRC5 | SESN3 |
| LGALS1 | REEP3 | C17orf77 |
| SRA1 | SMC4 | WIPF2 |
| CHI3L1 | CCNB1 | APCDD1 |
| MUL1 | NEDD1 | CTBP2 |
| NTN1 | FAM64A | ARL6IP6 |
| TRADD | CCNB2 | CNTN4 |
| BFAR | PHF13 | C4orf26 |
| PLK3 | RASSF1 | ZMAT2 |
| STEAP3 | CKS2 | ARSK |
| HINT1 | CUL4B | CYLC2 |
| DAXX | WTAP | RNF217 |
| PDCD2 | ANKLE2 | RLBP1L1 |
| RPS3 | CD2AP | GPR180 |
| TRIAP1 | PIK3C3 | RHBDL3 |
| CASP3 | CCNA2 | KANK4 |
| CASP7 | CIB1 | FAM43B |
| HMOX1 | LIG1 | C20orf75 |
| TNIP2 | RBL1 | DDX6 |
| CASP1 | RINT1 | BBS12 |
| NOL3 | PIM1 | GLIS3 |
| DAPK3 | PPP1CB | SPIN3 |
| TNFRSF10B | NCAPD3 | COMMD6 |
| BNIP2 | NCAPD2 | NUDT10 |
| THOC6 | MAPK1 | ADAMTS18 |
| BUB1B | PPP1CA | ABHD3 |
| MFSD10 | TIMELESS | DLX2 |
| PPP1R15A | CDCA7L | AGA |
| C3ORF38 | BIN3 | DOCK3 |
| THOC1 | MAPK7 | JAG1 |
| GPR65 | MAD2L2 | DSC3 |
| PML | NEK6 | SLC26A2 |
| TRIB3 | RAD17 | EDG3 |
| TNFRSF1A | EIF2AK4 | EGR3 |
| TNFRSF1B | GNAI3 | UBR1 |
| SH3GLB1 | STK10 | ELF1 |
| XBP1 | NEDD9 | ELF2 |
| SHISA5 | AHCTF1 | APOBEC3F |
| PYCARD | CEP164 | EMP1 |
| TNFRSF19 | ARF6 | UNC13D |
| PHLDA1 | KATNA1 | ZBTB7C |
| AIMP2 | FBXO5 | EP300 |
| SAP30BP | HELLS | HIPK1 |
| BIRC3 | TFDP1 | ETF1 |
| BIRC2 | CDC23 | F2R |
| APAF1 | CDC20 | F13A1 |
| DRAM1 | REEP4 | BPTF |
| IGFBP3 | PLK3 | HNRPA3 |
|  | SMC1A | OPN5 |
|  | PHF8 | GPR116 |
|  | KCTD11 | SEMA3D |
|  | STEAP3 | TFEC |
|  | E2F3 | ATF5 |
|  | E2F6 | KIAA0831 |
|  | E2F7 | ENPP4 |
|  | MITD1 | DIS3 |
|  | LATS2 | MAPRE1 |
|  | ZNF207 | SEPHS1 |
|  | RPS3 | UNC13A |
|  | NDE1 | FOXE1 |
|  | MCM7 | ENDOD1 |
|  | MASTL | NMNAT2 |
|  | TPR | ZNF609 |
|  | KHDRBS1 | SETX |
|  | CCNK | TXNDC4 |
|  | RBBP4 | MYCBP2 |
|  | NUSAP1 | SIPA1L3 |
|  | DYNLT1 | FRMD4B |
|  | BANP | TBC1D9 |
|  | CDK7 | RTF1 |
|  | RB1 | KIAA1024 |
|  | ECT2 | ANKRD12 |
|  | CDK2 | CAMTA1 |
|  | RAD50 | TSPYL4 |
|  | MCM5 | CAMSAP1L1 |
|  | CDK3 | DNAJC13 |
|  | MCM6 | SR140 |
|  | SASS6 | CRB1 |
|  | FANCD2 | RYBP |
|  | BUB1B | GPR161 |
|  | CHFR | CBX7 |
|  | GADD45A | POFUT1 |
|  | CKS1B | ZNF281 |
|  | HAUS3 | CD2AP |
|  | TRIOBP | C9orf5 |
|  | HAUS5 | FLRT2 |
|  | USP3 | FXN |
|  | HAUS2 | ALOX12B |
|  | HAUS1 | ALPL |
|  | CDC73 | FNDC5 |
|  | MDC1 | SRD5A2L2 |
|  | USP37 | C8orf46 |
|  | ZWILCH | ZDHHC23 |
|  | NSUN2 | FAM26C |
|  | CSNK1A1 | C3orf43 |
|  | BOD1 | GABRG1 |
|  | MIS12 | UNC84B |
|  | SON | RIPK5 |
|  | RPS6KA3 | GCA |
|  | PTP4A1 | NIPBL |
|  | CHTF18 | GALNT1 |
|  |  | SGMS1 |
|  |  | NALCN |
|  |  | SOSTDC1 |
|  |  | GANC |
|  |  | CHMP2B |
|  |  | GAPVD1 |
|  |  | KIF26A |
|  |  | ARL5A |
|  |  | TIAM2 |
|  |  | FBXL4 |
|  |  | GATA2 |
|  |  | CNNM1 |
|  |  | GHR |
|  |  | FOXD3 |
|  |  | MLH3 |
|  |  | FOXP1 |
|  |  | TAF5L |
|  |  | CACYBP |
|  |  | DKK2 |
|  |  | AHDC1 |
|  |  | PCDH17 |
|  |  | CECR6 |
|  |  | GNA12 |
|  |  | GNRHR |
|  |  | CDH19 |
|  |  | WDR21B |
|  |  | COX18 |
|  |  | GPR37 |
|  |  | OSTM1 |
|  |  | TMEM14A |
|  |  | N6AMT1 |
|  |  | C16orf80 |
|  |  | GTF2H1 |
|  |  | SEC61A1 |
|  |  | PDZRN4 |
|  |  | NRBF2 |
|  |  | SH3KBP1 |
|  |  | KCNIP3 |
|  |  | UBE2K |
|  |  | HLA-DQA1 |
|  |  | HMGB1 |
|  |  | HOXC13 |
|  |  | HPGD |
|  |  | XIAP |
|  |  | SPOPL |
|  |  | CYP27C1 |
|  |  | KIAA2022 |
|  |  | LHFPL1 |
|  |  | IGF1 |
|  |  | NUP43 |
|  |  | IL12B |
|  |  | IL13RA1 |
|  |  | ITPR2 |
|  |  | FAM111B |
|  |  | KCNK1 |
|  |  | KIF5C |
|  |  | C6orf120 |
|  |  | SBK1 |
|  |  | LOC388335 |
|  |  | VGLL3 |
|  |  | ZNF793 |
|  |  | LBR |
|  |  | LMNA |
|  |  | LMO7 |
|  |  | KLHL31 |
|  |  | PPAPDC2 |
|  |  | ZBTB34 |
|  |  | SMAD4 |
|  |  | MAF |
|  |  | MAN1A1 |
|  |  | MAP2 |
|  |  | MBP |
|  |  | MEFV |
|  |  | MFAP4 |
|  |  | MMP19 |
|  |  | PPP1R12A |
|  |  | MYT1 |
|  |  | NAP1L4 |
|  |  | NEDD4 |
|  |  | NEUROD2 |
|  |  | NF1 |
|  |  | NFE2L2 |
|  |  | NFIX |
|  |  | NFKB1 |
|  |  | NPAT |
|  |  | NPY2R |
|  |  | LOC493869 |
|  |  | PAK2 |
|  |  | DDEF1 |
|  |  | TRAT1 |
|  |  | SPOCK3 |
|  |  | PBX3 |
|  |  | ISOC1 |
|  |  | NMD3 |
|  |  | FAM82B |
|  |  | C1RL |
|  |  | NRN1 |
|  |  | TAOK3 |
|  |  | PDE1A |
|  |  | CRLF3 |
|  |  | FAM8A1 |
|  |  | CTDSPL2 |
|  |  | DTL |
|  |  | GDE1 |
|  |  | KLF13 |
|  |  | OTUD6B |
|  |  | PDPK1 |
|  |  | C9orf78 |
|  |  | TM7SF3 |
|  |  | PF4V1 |
|  |  | PGR |
|  |  | ABCB1 |
|  |  | PIK3R1 |
|  |  | PITX1 |
|  |  | PKD2 |
|  |  | PKHD1 |
|  |  | PKNOX1 |
|  |  | ATP7A |
|  |  | KIAA1128 |
|  |  | FAM134B |
|  |  | TXNDC10 |
|  |  | RBM47 |
|  |  | POU2F2 |
|  |  | CCDC93 |
|  |  | POU3F1 |
|  |  | ING3 |
|  |  | FAM63B |
|  |  | RSBN1 |
|  |  | BNC2 |
|  |  | KLHL24 |
|  |  | SAMD9 |
|  |  | KLHL28 |
|  |  | ANKRD49 |
|  |  | MS4A12 |
|  |  | TBC1D8B |
|  |  | UBE2R2 |
|  |  | C1orf123 |
|  |  | USP47 |
|  |  | PTCD3 |
|  |  | C9orf40 |
|  |  | SDAD1 |
|  |  | ARHGEF10L |
|  |  | TYW1 |
|  |  | FBXW7 |
|  |  | PPP3CA |
|  |  | PHCA |
|  |  | YOD1 |
|  |  | CAMK2N1 |
|  |  | IL17RB |
|  |  | THUMPD1 |
|  |  | CHD7 |
|  |  | PRKAB2 |
|  |  | SLC30A6 |
|  |  | PKIB |
|  |  | PRKCB1 |
|  |  | PRKCE |
|  |  | PAG1 |
|  |  | ZNF395 |
|  |  | NXT2 |
|  |  | MAPK1 |
|  |  | ANKH |
|  |  | CRTAM |
|  |  | BRUNOL4 |
|  |  | FSTL5 |
|  |  | SPIRE1 |
|  |  | TMEPAI |
|  |  | STOX2 |
|  |  | TULP4 |
|  |  | ACN9 |
|  |  | SALL4 |
|  |  | ATP8B2 |
|  |  | PTGFR |
|  |  | RAB22A |
|  |  | AS3MT |
|  |  | ARID1B |
|  |  | PCDH19 |
|  |  | NUFIP2 |
|  |  | WDR35 |
|  |  | KIAA1377 |
|  |  | ZNF471 |
|  |  | SLAIN2 |
|  |  | PTN |
|  |  | PHF12 |
|  |  | ZBTB4 |
|  |  | ALS2 |
|  |  | KIAA1600 |
|  |  | PTPN4 |
|  |  | RBAK |
|  |  | PTPN12 |
|  |  | C6orf47 |
|  |  | CADM3 |
|  |  | PTPRB |
|  |  | PTPRZ1 |
|  |  | RHOU |
|  |  | RAD51L1 |
|  |  | RARRES1 |
|  |  | ARID4A |
|  |  | RET |
|  |  | EDA2R |
|  |  | TGIF2 |
|  |  | BACH2 |
|  |  | SLC5A7 |
|  |  | ROCK1 |
|  |  | ZNF704 |
|  |  | RPS6KB1 |
|  |  | RYR2 |
|  |  | S100A2 |
|  |  | CXCL5 |
|  |  | XCL1 |
|  |  | SDC2 |
|  |  | C14orf133 |
|  |  | FAM38B |
|  |  | PRDM1 |
|  |  | PERP |
|  |  | PAPD5 |
|  |  | LMBR1 |
|  |  | SFRS10 |
|  |  | C5orf28 |
|  |  | C14orf135 |
|  |  | SHOX2 |
|  |  | MOSC1 |
|  |  | CREB3L2 |
|  |  | GINS3 |
|  |  | TBC1D15 |
|  |  | RFXDC2 |
|  |  | BCL11B |
|  |  | SIM1 |
|  |  | SIM2 |
|  |  | STAG3L4 |
|  |  | REEP1 |
|  |  | SLC1A2 |
|  |  | MRPL44 |
|  |  | ZBTB8 |
|  |  | SLC7A1 |
|  |  | BMPR2 |
|  |  | ZBTB10 |
|  |  | SMARCC1 |
|  |  | SNAPC3 |
|  |  | SNCA |
|  |  | SOX2 |
|  |  | SOX3 |
|  |  | SOX4 |
|  |  | TROVE2 |
|  |  | STAT3 |
|  |  | STAT5B |
|  |  | ZFP36L2 |
|  |  | STCH |
|  |  | STK3 |
|  |  | SULT2A1 |
|  |  | TACC1 |
|  |  | TAL1 |
|  |  | DYNLT3 |
|  |  | TERF2 |
|  |  | TIAL1 |
|  |  | NR2C2 |
|  |  | TRIO |
|  |  | TTK |
|  |  | LOC728215 |
|  |  | VCP |
|  |  | WNT5A |
|  |  | XRCC2 |
|  |  | YWHAB |
|  |  | ZFX |
|  |  | VEZF1 |
|  |  | ZNF208 |
|  |  | NPHS2 |
|  |  | SLMAP |
|  |  | SLBP |
|  |  | MRP63 |
|  |  | EPM2A |
|  |  | C6orf211 |
|  |  | C1orf108 |
|  |  | MANEA |
|  |  | GALNT12 |
|  |  | ANKRD55 |
|  |  | LIN28 |
|  |  | TXNDC15 |
|  |  | VASH2 |
|  |  | THSD4 |
|  |  | ATP8B4 |
|  |  | CALCR |
|  |  | POF1B |
|  |  | PLEKHH3 |
|  |  | FLJ13611 |
|  |  | GLRA3 |
|  |  | WWC2 |
|  |  | CALM1 |
|  |  | C6orf97 |
|  |  | NARG1 |
|  |  | MYCT1 |
|  |  | CXorf21 |
|  |  | LRRC27 |
|  |  | SHOC2 |
|  |  | CAMK4 |
|  |  | SYNC1 |
|  |  | PLA2G12A |
|  |  | SBF2 |
|  |  | CAMLG |
|  |  | SMC1A |
|  |  | ARHGAP24 |
|  |  | PUS3 |
|  |  | RASSF5 |
|  |  | AMMECR1L |
|  |  | CRISPLD1 |
|  |  | CASP6 |
|  |  | CTTNBP2 |
|  |  | FBXO30 |
|  |  | MLSTD2 |
|  |  | BTBD10 |
|  |  | WIBG |
|  |  | MEGF11 |
|  |  | CUL3 |
|  |  | ATRN |
|  |  | PARD6G |
|  |  | AFAP1L2 |
|  |  | N-PAC |
|  |  | ZNF514 |
|  |  | SPPL2A |
|  |  | TMTC4 |
|  |  | UBASH3B |
|  |  | CSDA |
|  |  | CBX4 |
|  |  | C4orf35 |
|  |  | UNK |
|  |  | AP3B1 |
|  |  | NCOA1 |
|  |  | EIF4G3 |
|  |  | CBLB |
|  |  | B3GALT1 |
|  |  | NOL4 |
|  |  | ADAM9 |
|  |  | BCL10 |
|  |  | BTBD6 |
|  |  | MAP7 |
|  |  | ANGPTL1 |
|  |  | DNAJA3 |
|  |  | CD1B |
|  |  | PRPF4 |
|  |  | XRCC6BP1 |
|  |  | DDX21 |
|  |  | SLC16A7 |
|  |  | OXNAD1 |
|  |  | DLGAP2 |
|  |  | PTER |
|  |  | TRIP13 |
|  |  | SNAP29 |
|  |  | LHX2 |
|  |  | ACTR8 |
|  |  | CRIPT |
|  |  | ZNF264 |
|  |  | TP53INP1 |
|  |  | MED26 |
|  |  | IL27RA |
|  |  | SH3BP5 |
|  |  | NPEPPS |
|  |  | EI24 |
|  |  | NRG2 |
|  |  | VPS26A |
|  |  | GABBR2 |
|  |  | PREPL |
|  |  | CREB5 |
|  |  | GDA |
|  |  | RIMS2 |
|  |  | USP6NL |
|  |  | KIAA0408 |
|  |  | PCDHA9 |
|  |  | TOX |
|  |  | ACYP2 |
|  |  | TOMM20 |
|  |  | MFAP3L |
|  |  | LRIG2 |
|  |  | FCHSD2 |
|  |  | ZBTB5 |
|  |  | AMMECR1 |
|  |  | GOLGA5 |
|  |  | USP15 |
|  |  | APPBP2 |
|  |  | ARL6IP5 |
|  |  | ADCY2 |
|  |  | E2F7 |
|  |  | ZXDB |
|  |  | FGF12 |
|  |  | CPEB3 |
|  |  | ATP10B |
|  |  | FMR1 |
|  |  | MYST4 |
|  |  | CADM1 |
|  |  | LRIG1 |
|  |  | AUTS2 |
|  |  | GFRA2 |
|  |  | TSPAN13 |
|  |  | RBMS3 |
|  |  | LYPD5 |
|  |  | XKR6 |
|  |  | ANK3 |
|  |  | ICAM4 |
|  |  | MTR |
|  |  | MYO9A |
|  |  | NDUFA10 |
|  |  | NFIB |
|  |  | LOC492311 |
|  |  | NR4A2 |
|  |  | OCRL |
|  |  | AADAT |
|  |  | HEMK1 |
|  |  | AMOTL2 |
|  |  | SERPINB13 |
|  |  | C10orf18 |
|  |  | FAM46A |
|  |  | C12orf11 |
|  |  | ANKS1B |
|  |  | OTUD7B |
|  |  | SLC17A6 |
|  |  | BCHE |
|  |  | CPLX3 |
|  |  | GIGYF1 |
|  |  | SLC6A2 |
|  |  | SLC13A1 |
|  |  | FLJ11506 |
|  |  | MAP6D1 |
|  |  | PGAP1 |
|  |  | TET1 |
|  |  | RGS5 |
|  |  | SLC43A1 |
|  |  | PAQR8 |
|  |  | MCFD2 |
|  |  | NRXN2 |
|  |  | DNAJC6 |
|  |  | ZEB2 |
