# Supplementary material for: DNA-methylation-mediated activating of lncRNA SNHG12 promotes temozolomide resistance in glioblastoma
Source: Mol Cancer. 2020 Feb 10;19:28. doi: 10.1186/s12943-020-1137-5 (PMC7011291; doi:10.1186/s12943-020-1137-5)
Supplement: Supplementary file 6 — Additional file 6: Figure S1. SNHG12 is up-regulated in GBM, related to Fig. 1. [file 12943_2020_1137_MOESM6_ESM.docx]

**Figure S1**


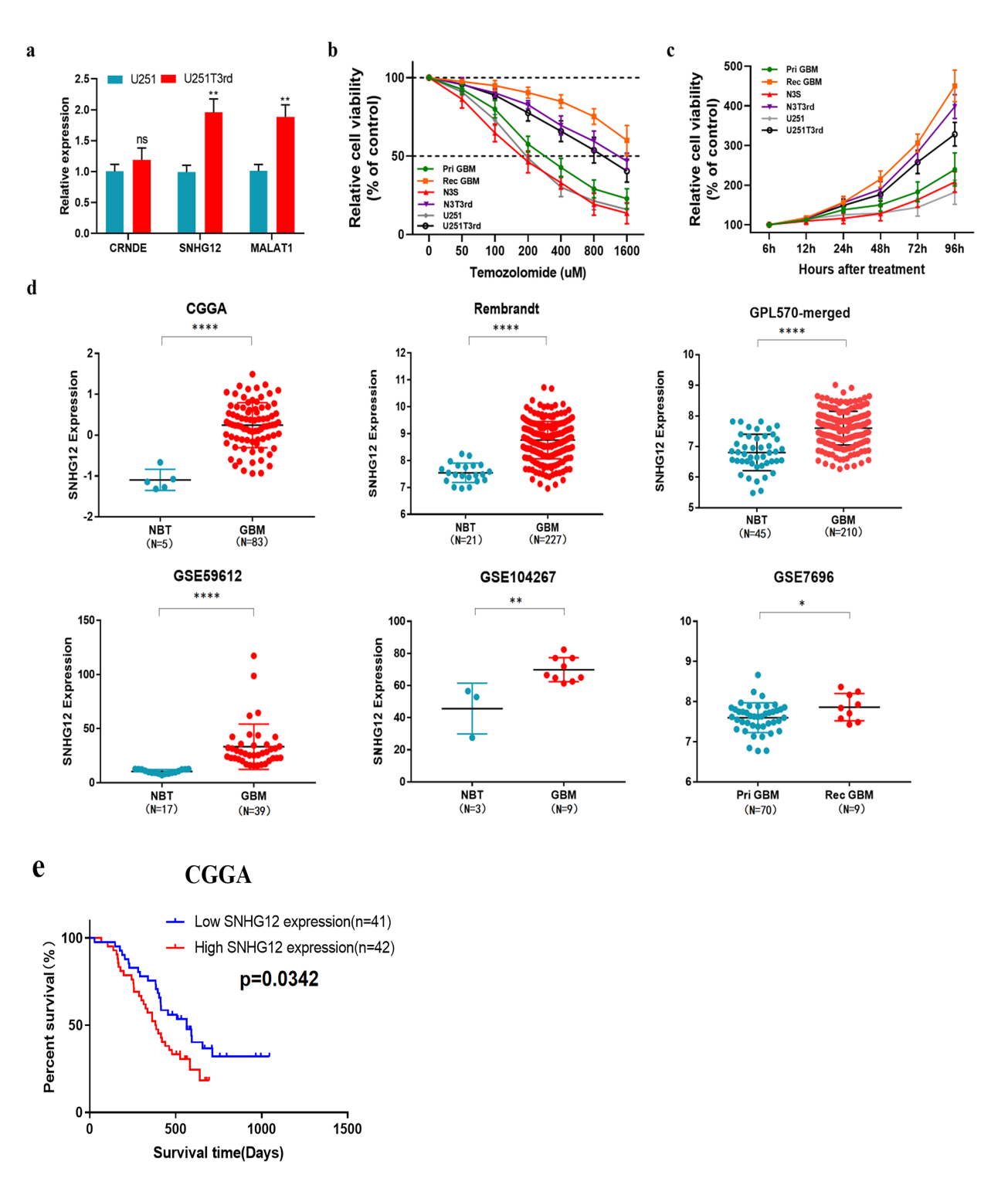


**Figure S1 SNHG12 is up-regulated in GBM, related to Fig. 1**

**a** The expression level of SNHG12 in U251 and U251T3rd cells. **b** CCK8 assay was used to evaluate cell proliferation ability of 6 GBM cells after 48 h TMZ treatment. **c** Cell proliferation was evaluated in 6 GBM cells after 200 μM TMZ treatments using CCK8 assay. **d** Expression of SNHG12 in the CGGA, Rembrandt, GPL570-merged (GSE4290, GSE7696, GSE15824, GSE50161), GSE59612, GSE104267, GSE7696 data sets. **e** Kaplan-Meier survival plots demonstrating that SNHG12 expression levels related to worse OS in GBM patients in CGGA data set (n = 83, P = 0.0342). Data are presented as the mean ± SEM from three independent experiments. Significant results were presented as NS non-significant , **P*＜0.05, ***P*＜0.01, *****P*＜0.0001.
