# Supplementary material for: DNA-methylation-mediated activating of lncRNA SNHG12 promotes temozolomide resistance in glioblastoma
Source: Mol Cancer. 2020 Feb 10;19:28. doi: 10.1186/s12943-020-1137-5 (PMC7011291; doi:10.1186/s12943-020-1137-5)
Supplement: Supplementary file 7 — Additional file 7: Figure S2. SNHG12 levels correlate with temozolomide resistance, related to Figs. 2-3. [file 12943_2020_1137_MOESM7_ESM.docx]

**Figure S2**

**
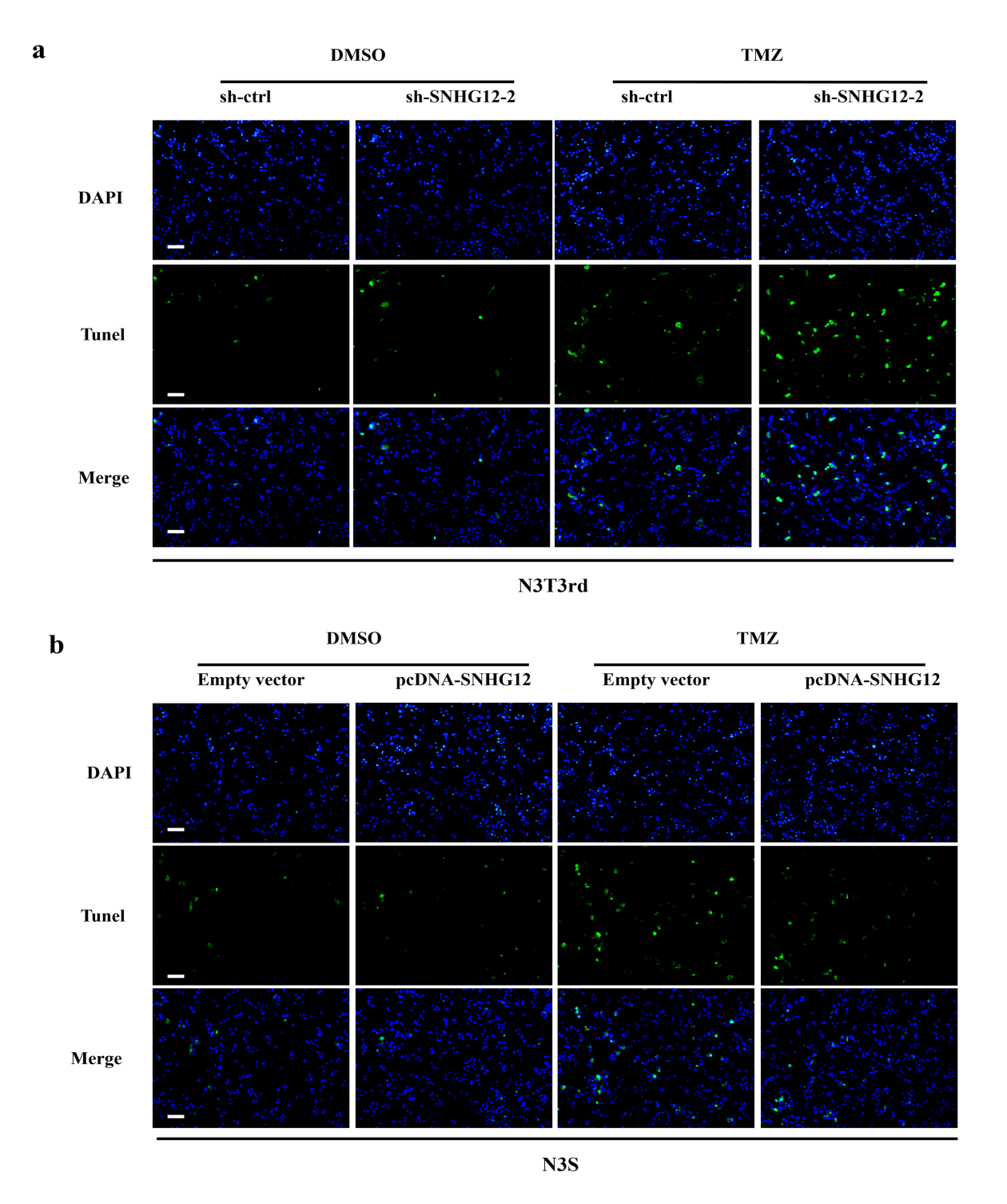
**

**Figure S2 SNHG12 levels correlate with temozolomide resistance, related to Fig. 2-3**

**a** TUNEL analysis of SNHG12 knockdown cells or vehicle control with or without TMZ treatment (200 μM, 48 h). Scale bar = 50μm. **b** TUNEL analysis of SNHG12 overexpression cells or empty vector with or without TMZ treatment (200 μM, 48 h). Scale bar = 50μm.
