# Supplementary material for: DNA-methylation-mediated activating of lncRNA SNHG12 promotes temozolomide resistance in glioblastoma
Source: Mol Cancer. 2020 Feb 10;19:28. doi: 10.1186/s12943-020-1137-5 (PMC7011291; doi:10.1186/s12943-020-1137-5)
Supplement: Supplementary file 8 — Additional file 8: Figure S3. DNA methylation and SP1 regulate SNHG12 expression level, related to Fig. 4. [file 12943_2020_1137_MOESM8_ESM.docx]

**Figure S3**


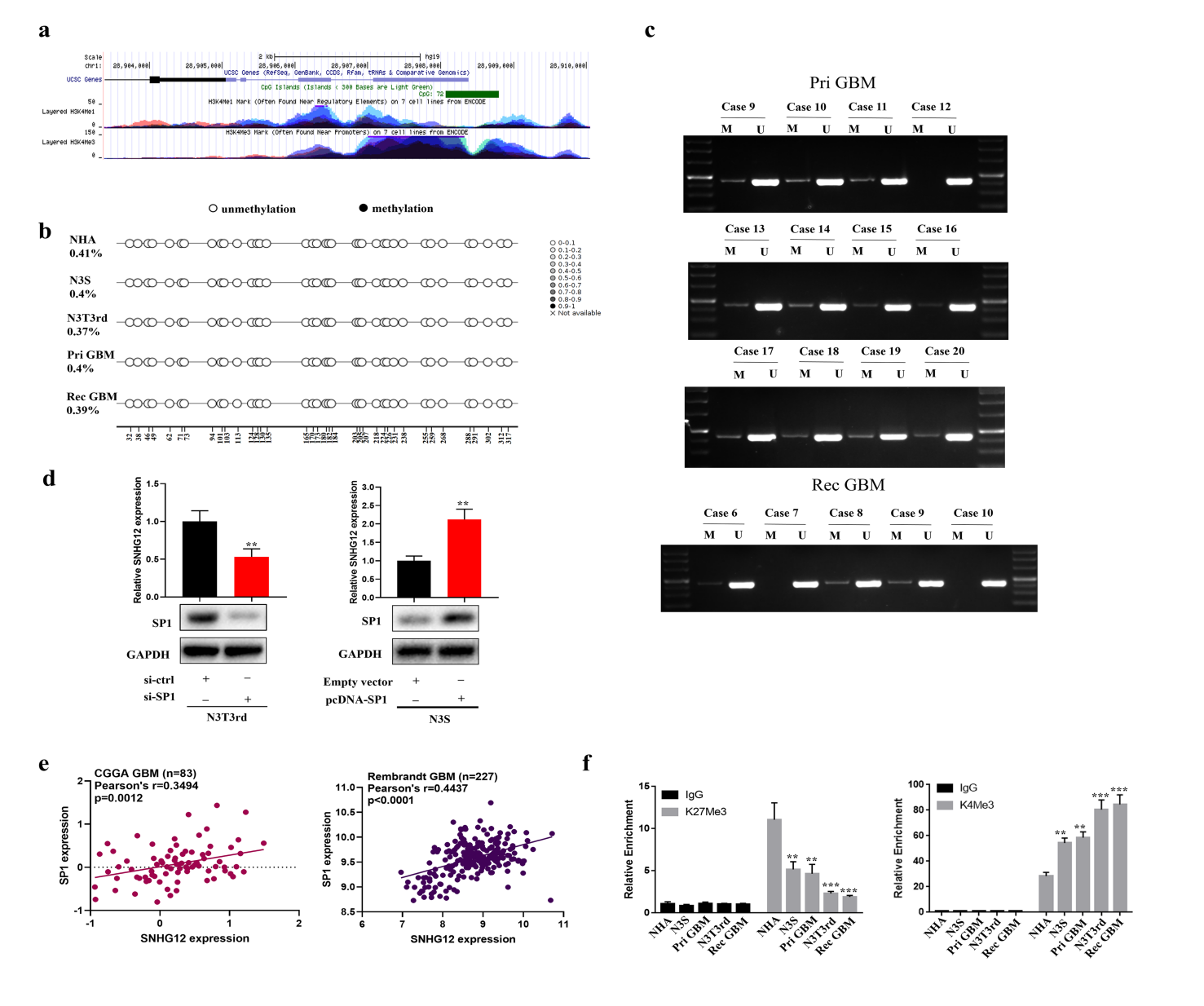


**Figure S3 DNA methylation and SP1 regulate SNHG12 expression level, related to Fig. 4**

**a** The UCSC Genome Bioinformatics Site (http://genome.ucsc.edu/) showed high enrichment of H3K4me1 and H3K4me3 at the promoter of SNHG12. **b** Bisulfite genomic sequencing was performed to examine methylation status of CpG island 2 at the promoter region of SNHG12 in NHA, Pri GBM, Rec GBM, N3S, N3T3rd cells. **c** MSP analysis was performed to examine methylation status of CpG island 1 at the promoter region of SNHG12 in primary GBM tissues and recurrent GBM tissues. **d** The SNHG12 levels were detected in N3T3rd and N3S cells either stably expressing SP1 or with SP1 depleted. **e** The correlation between SP1 and SNHG12 in GBM tissues in CGGA and Rembrandt data sets was analyzed. **f** ChIP analysis for the detection of the presence H3K27Me^3^ and H3K4Me^3^ at the promoter region of SNHG12. Data are presented as the mean ± SEM from three independent experiments. Significant results were presented as **P＜0.01.
