# Supplementary material for: DNA-methylation-mediated activating of lncRNA SNHG12 promotes temozolomide resistance in glioblastoma
Source: Mol Cancer. 2020 Feb 10;19:28. doi: 10.1186/s12943-020-1137-5 (PMC7011291; doi:10.1186/s12943-020-1137-5)
Supplement: Supplementary file 9 — Additional file 9: Figure S4. SNHG12 act as a sponge for miR-129-5p in the cytoplasm, related to Fig. 5. [file 12943_2020_1137_MOESM9_ESM.docx]

**Figure S4**


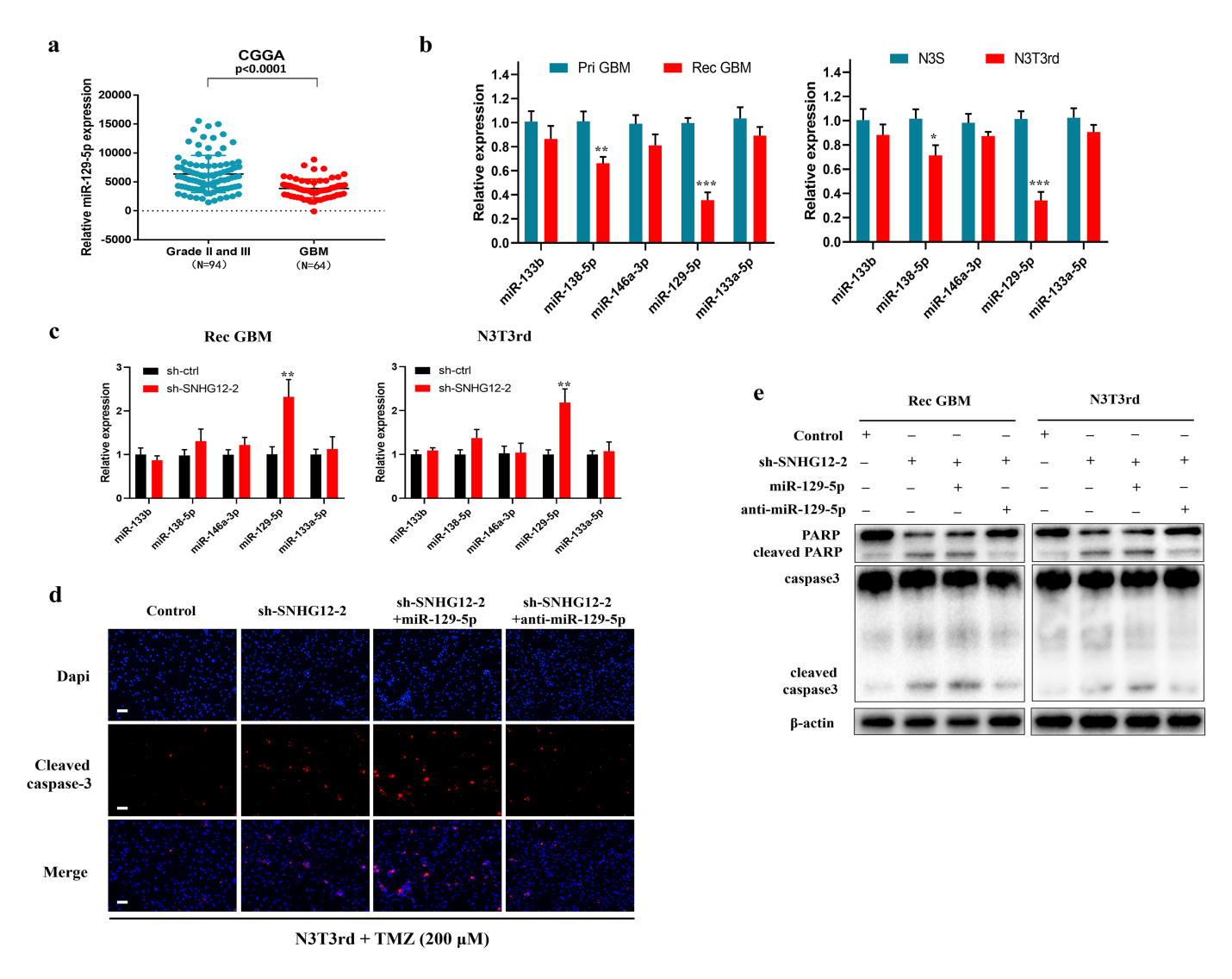


**Figure S4 SNHG12 act as a sponge for miR-129-5p in the cytoplasm, related to Fig. 5**

**a** The expression level of miR-129-5p in CGGA data set. **b** The expression level of 5 predicted miRNAs in TMZ-sensitive and TMZ-resistant cells. **c** After transfected with sh-ctrl or sh-SNHG12 in Rec GBM and N3T3rd cells, the expression level of 5 predicted miRNAs was analyzed using real-time PCR. **d** Immunofluorescent staining of cleaved caspase-3 in N3T3rd cells transfected with SNHG12 plasmid, miR-129-5p mimics + SNHG12 plasmid or miR-129-5p inhibitor + SNHG12 plasmid after 200 μM TMZ treatment for 48h. Scale bar = 50μm. **e** Western blot test of caspase-3 and PARP in TMZ-resistant cells treated with vehicle control or TMZ (200 μM) for 48 h. β-actin was used as the loading control. Data are presented as the mean ± SEM from three independent experiments. Significant results were presented as NS non-significant, **P*＜0.05, ***P*＜0.01, ****P*＜0.001, *****P*＜0.0001.
