# Supplementary material for: DNA-methylation-mediated activating of lncRNA SNHG12 promotes temozolomide resistance in glioblastoma
Source: Mol Cancer. 2020 Feb 10;19:28. doi: 10.1186/s12943-020-1137-5 (PMC7011291; doi:10.1186/s12943-020-1137-5)
Supplement: Supplementary file 10 — Additional file 10: Figure S5. SNHG12 regulates MAPK1 and E2F7 expression by competitively binding miR-129-5p, related to Fig. 6 [file 12943_2020_1137_MOESM10_ESM.docx]

**Figure S5**


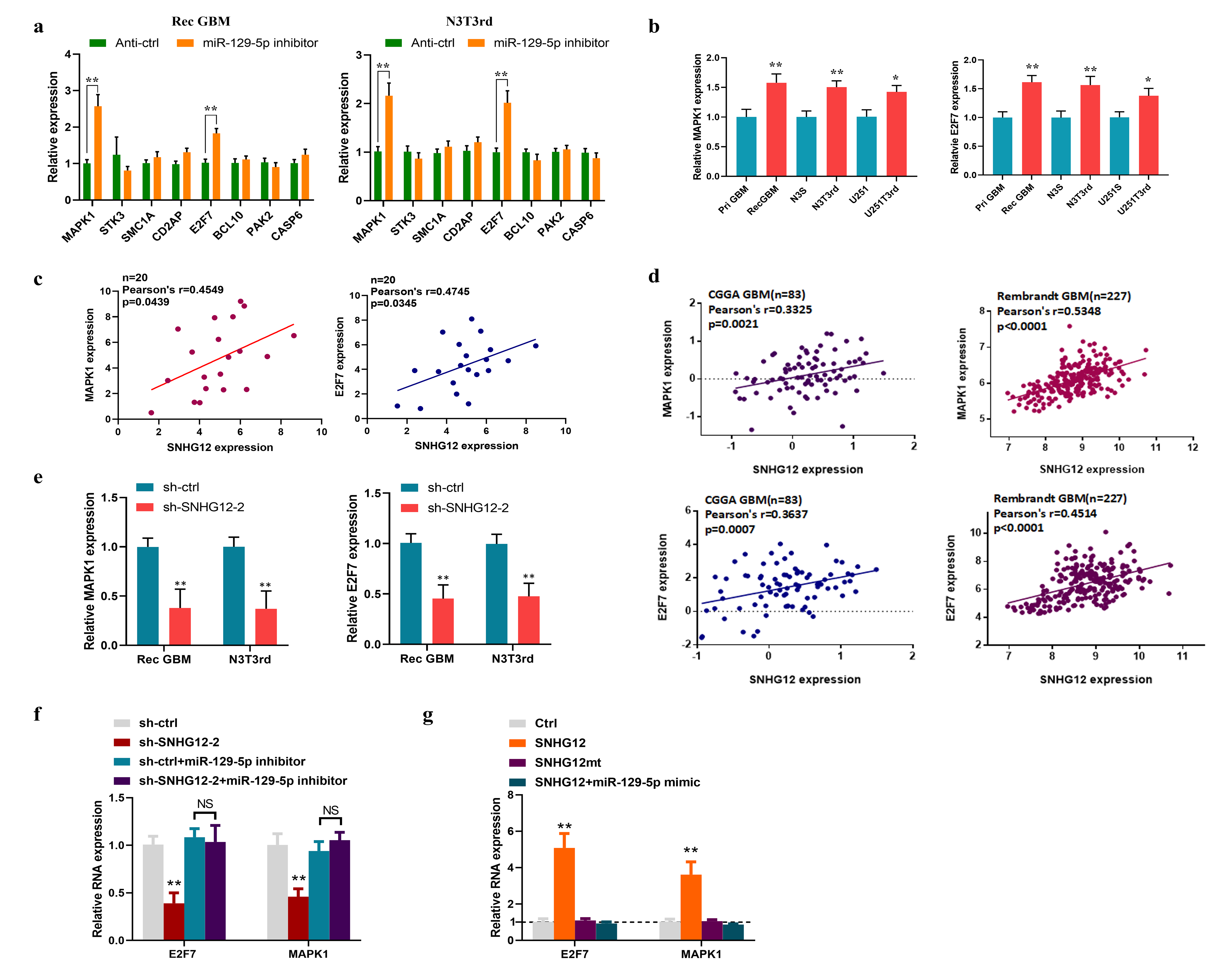


**Figure S5 SNHG12 regulates MAPK1 and E2F7 expression by competitively binding miR-129-5p, related to Fig. 6**

**a** After transfected with miR-129-5p inhibitor in Rec GBM and N3T3rd cells, the expression level of 8 potential targets for miR-129-5p was analyzed using real-time PCR. **b** The expression level of MAPK1 and E2F7 in TMZ-sensitive and TMZ-resistant cells. **c** Right: Association analysis of the relationship between SNHG12 and E2F7 expression levels in 20 recurrent GBM tissues. Left: Association analysis of the relationship between SNHG12 and MAPK1 expression levels in 20 recurrent GBM tissues. **d** The correlation between MAPK1 and SNHG12 , E2F7 and SNHG12 in GBM tissues in CGGA and Rembrandt data sets. **e** The expression level of MAPK1 and E2F7 in Rec GBM and N3T3rd cells after transfected with sh-ctrl or sh-SNHG12. **f** qRT-PCR analysis of E2F7 and MAPK1 in Rec GBM cells and N3T3rd cells transfected with control or SNHG12 shRNA along with miR-129-5p inhibitor. **g** qRT-PCR analysis of E2F7 and MAPK1 in Pri GBM cells and N3S cells transfected pcDNA-SNHG12 or pcDNA-SNHG12mt along with indicated miR-129-5p mimics. Data are presented as the mean ± SEM from three independent experiments. Significant results were presented as NS non-significant, **P*＜0.05, ***P*＜0.01.
