# Supplementary material for: DNA-methylation-mediated activating of lncRNA SNHG12 promotes temozolomide resistance in glioblastoma
Source: Mol Cancer. 2020 Feb 10;19:28. doi: 10.1186/s12943-020-1137-5 (PMC7011291; doi:10.1186/s12943-020-1137-5)
Supplement: Supplementary file 11 — Additional file 11: Figure S6. SNHG12 accelerates temozolomide resistance in GBM cells via MAPK1 and E2F7, related to Fig. 7. [file 12943_2020_1137_MOESM11_ESM.docx]

**Figure S6**


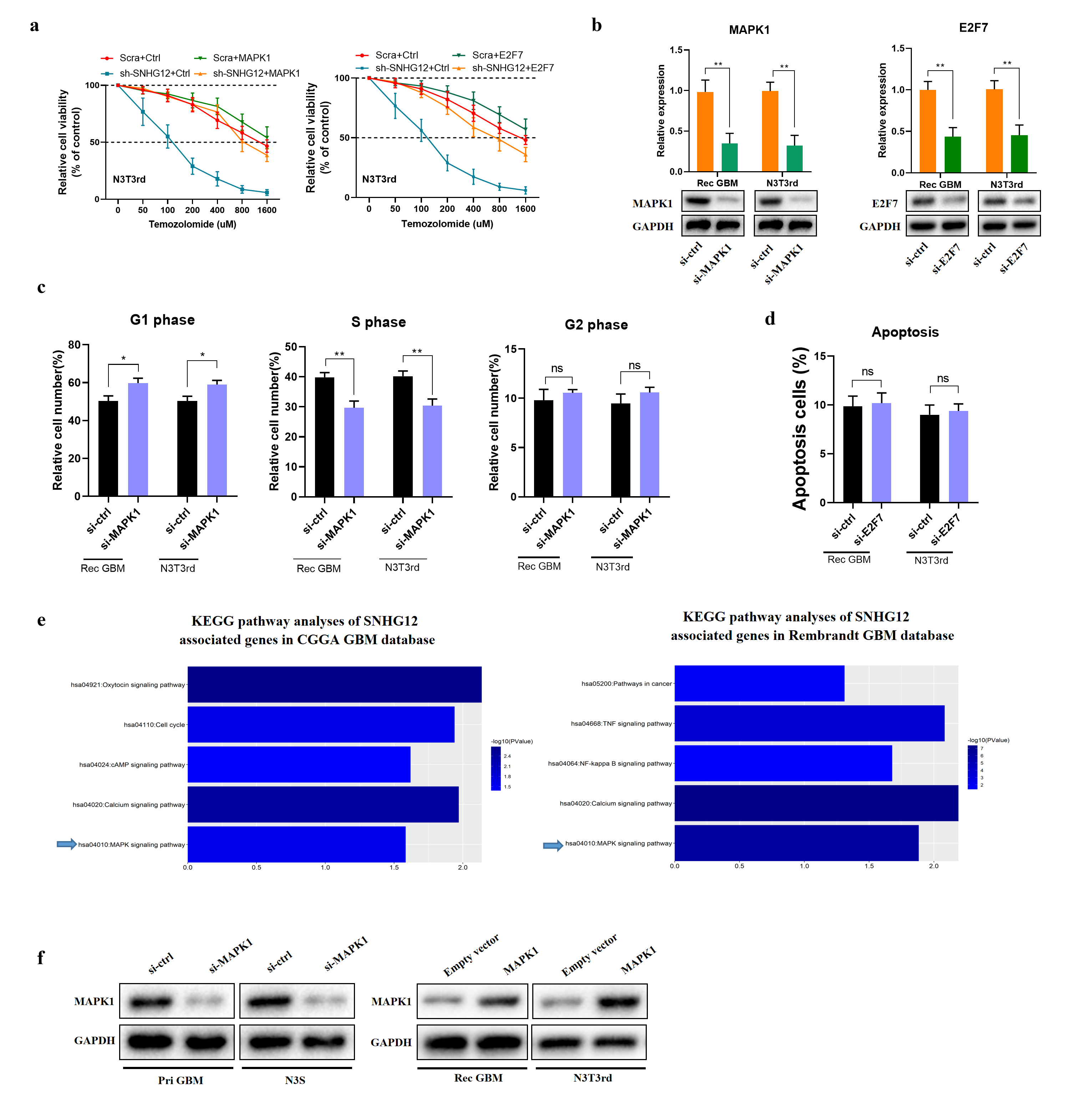


**Figure S6 SNHG12 accelerates temozolomide resistance in GBM cells via MAPK1 and E2F7, related to Fig. 7**

**a** CCK-8 assay analysis of the effect of MAPK1 or E2F7 overexpression on N3T3rd cells after knocking down SNHG12 upon TMZ treatment at the indicated concentrations for 48h. **b** The results of western blotting and real-time PCR showing the knockdown of MAPK1 or E2F7 in Rec GBM and N3T3rd cells by transfection of specific siRNAs. **c** Dysregulation of MAPK1 in Rec GBM and N3T3rd cells affected the G1/S cell cycle transition. **d** Dysregulation of E2F7 has no effect on cell apoptosis. **e** Pathway analyses were performed using the SNHG12 associated genes in CGGA and Rembrandt data sets. **f** The MAPK1 protein levels were determined by western blot in GBM cells. Data are presented as the mean ± SEM from three independent experiments. Significant results were presented as NS non-significant, **P*＜0.05, ***P*＜0.01.
